# Cell Growth of Wall-Free L-Form Bacteria Is Limited by Oxidative Damage

**DOI:** 10.1016/j.cub.2015.04.031

**Published:** 2015-06-15

**Authors:** Yoshikazu Kawai, Romain Mercier, Ling Juan Wu, Patricia Domínguez-Cuevas, Taku Oshima, Jeff Errington

**Affiliations:** 1 Centre for Bacterial Cell Biology, Institute for Cell and Molecular Biosciences, Medical School, Newcastle University, Richardson Road, Newcastle upon Tyne NE2 4AX, UK; 2 Department of Biology, University of Copenhagen, Ole Maaløes Vej 5, 2200 Copenhagen N, Denmark; 3 Genomics of Bacterial Cell Functions, Graduate School of Biological Sciences, Nara Institute of Science and Technology, 8916-5, Takayama, Ikoma, Nara 630-0192, Japan

## Abstract

The peptidoglycan (PG) cell wall is a defining feature of the bacterial lineage and an important target for antibiotics, such as β-lactams and glycopeptides. Nevertheless, many bacteria are capable of switching into a cell-wall-deficient state, called the “L-form” [1–3]. These variants have been classically identified as antibiotic-resistant forms in association with a wide range of infectious diseases [4]. L-forms become completely independent of the normally essential FtsZ cell division machinery [3, 5]. Instead, L-form proliferation is driven by a simple biophysical process based on an increased ratio of surface area to cell volume synthesis [6, 7]. We recently showed that only two genetic changes are needed for the L-form transition in *Bacillus subtilis* [7]. Class 1 mutations work to generate excess membrane synthesis [7]. Until now, the function of the class 2 mutations was unclear. We now show that these mutations work by counteracting an increase in the cellular levels of reactive oxygen species (ROS) originating from the electron transport pathway, which occurs in wall-deficient cells. Consistent with this, addition of a ROS scavenger or anaerobic culture conditions also worked to promote L-form growth without the class 2 mutations in both Gram-positive *B*. *subtilis* and Gram-negative *Escherichia coli*. Our results suggest that physiological compensation for the metabolic imbalance that occurs when cell wall synthesis is blocked is crucial for L-form proliferation in a wide range of bacteria and also provide new insights into the mode of action of antibiotics that target the bacterial cell wall.

## Highlights

• The cellular levels of ROS are increased when cell wall synthesis is blocked
• Oxidative damage is a serious impediment to growth of wall-deficient L-forms
• Reduction of ROS levels promotes L-form growth
• L-forms provide new insights into the mode of action of cell wall antibiotics

## Teaser

Although many bacteria are capable of switching into a cell-wall-deficient L-form state, the molecular basis of the transition is still poorly understood. Kawai et al. show that the cellular levels of ROS are abnormally increased when cell wall synthesis is blocked and that counteraction of ROS production leads to improved L-form growth.

## Results

### The *ispA* Mutation Suppresses Cell Lysis during Protoplast Growth

**Figure 1:**
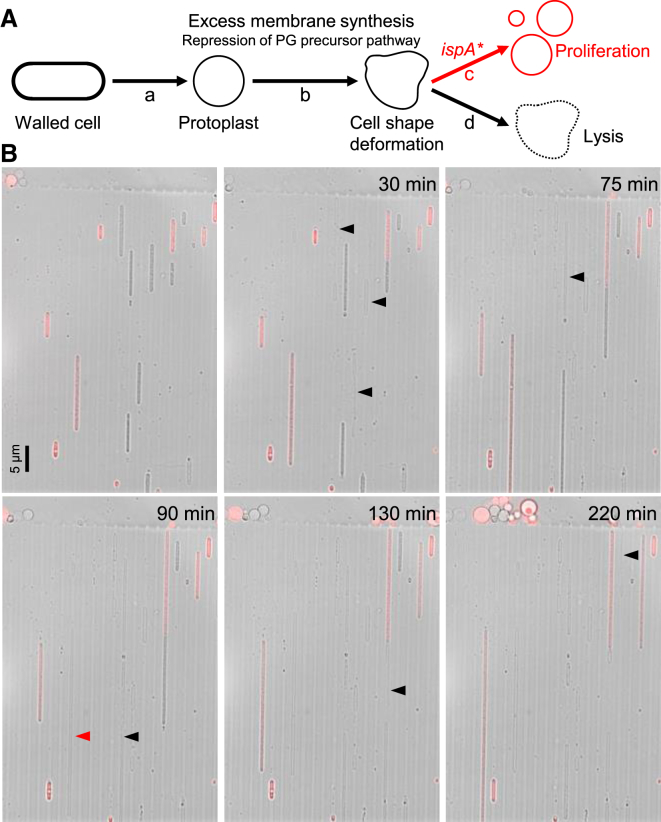
Effects of *ispA* Mutation and Repression of PG Precursor Pathway in Protoplast Growth (A) Schematic representation of the requirements for cell proliferation in cell-wall-free *B*. *subtilis*. See text for details. (B) *B*. *subtilis* strains, BS115 (*P*_*xyl*_-*murE*) and 4738 (*P*_*xyl*_-*murE ispA^∗^ aprE*::*P*_*rpsD*_*-mcherry*), were grown in the walled state (with xylose), then converted to protoplasts, and incubated in nutrient broth (NB) and magnesium-sucrose-maleic acid (MSM) (no xylose) with PenG. After 5 hr, the cultures were mixed, and the cells were observed by time-lapse microscopy via a microfluidic system. Representative images are shown of overlays of the phase contrast (PC) and corresponding mCherry signals. Elapsed time (min) is shown in each panel. Lysed cells are labeled with arrowheads. The scale bar represents 5 μm. See also Movie S1.

We previously showed that protoplasts of *B. subtilis*, derived by stripping the cell wall with lysozyme in the presence of an osmoprotective agent such as sucrose (Figure 1A, a), fail to proliferate in this state [7]. However, mutants able to proliferate, called “L-forms,” can be selected, and this requires a combination of two kinds of mutations [5, 7]. Class 1 mutations are of several different types, but all work by generating excess amounts of cell membrane, which drives spontaneous shape changes and ultimately proliferation (Figure 1A, b). The original class 2 mutation, which lay in a gene called *ispA* [5], seemed to work by stabilizing the proliferating L-forms and preventing them from lysing [7] (Figure 1A, c and d). For reasons that remain unclear, mutations that block cell wall precursor synthesis have a class 1 phenotype (i.e., generate excess cell membrane), and this is convenient because these mutations simultaneously prevent the cell wall from being restored.

To improve our understanding of the effects of the *ispA* mutation on cell lysis, we took advantage of recently developed microfluidic methods [8]. In channels of the microfluidic system, protoplasts are constrained into a near-typical rod-shaped morphology, with approximately similar width to that of wild-type walled cells. Figure 1B (and Movie S1) shows a mixture of *B*. *subtilis* protoplasts containing a repressible *P*_*xyl*_*-murE* construct (acts as a class 1 mutation) with (*ispA*^−^; red cells expressing mCherry) or without (*ispA*^+^; unlabelled cells) an *ispA* mutation trapped in the channels. The *ispA*^−^ mutant protoplasts (red cells) mainly grew well over many hours in L-form medium in the absence of xylose. In contrast, *ispA*^+^ protoplasts (unlabelled cells) frequently lysed after only a limited amount of growth (94% of *ispA*^+^ [n = 36] and 35% of *ispA*^−^ protoplasts [n = 23] resulted in cell lysis in similar experiments). Thus, protoplasts in which peptidoglycan (PG) synthesis is inhibited tend to lyse even in the absence of L-form-like shape changes and cell division, and this cell lysis is suppressed by an *ispA* mutation.

### Reduction of Electron Transport Chain Activity Promotes L-Form Growth

**Figure 2:**
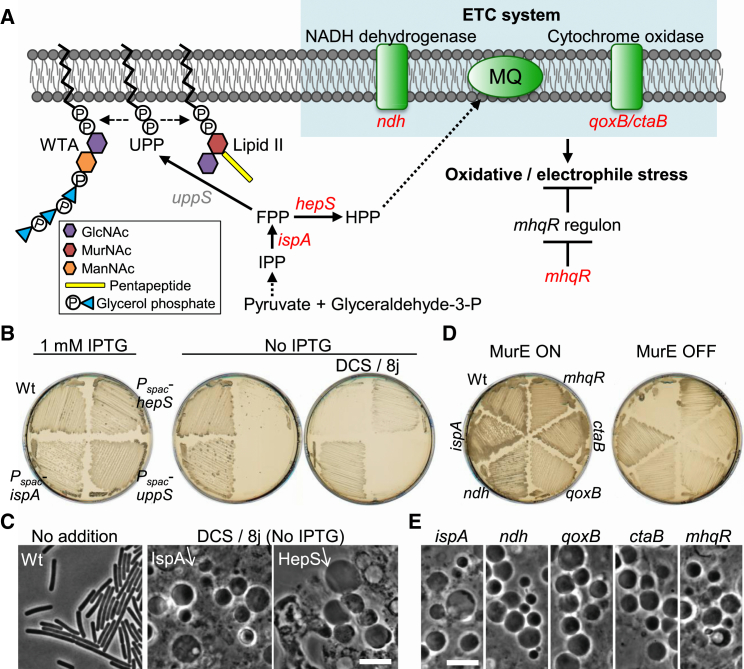
Effects of ETC Activity on L-Form Growth (A) Schematic representation of the UPP and HPP synthetic pathways and ETC system in *B*. *subtilis*. Repression of various genes (indicated in red) supports L-form growth when combined with inhibition of the PG precursor pathway. See text for details. (B) Effects of repression of *ispA*, *uppS*, and *hepS* on cell growth in the absence of a cell wall. *B*. *subtilis* strains, wild-type (Wt; 168CA), YK1424 (*P*_*spac*_-*ispA*), YK1889 (*ΔuppS* pLOSS-*P*_*spac*_-*uppS P*_*xyl*_-*cdsA*), and YK1450 (*P*_*spac*_-*hepS*) were grown on nutrient agar (NA) and MSM plates containing 1% xylose with or without 1 mM IPTG and 400 μg/ml DCS (with 1 μg/ml 8j to prevent the rare reversion to walled cells) at 30°C. Note that the *uppS* lies immediately upstream of *cdsA*, which is essential for membrane phospholipid synthesis. To avoid the polar effect on the *cdsA* expression, we inserted a xylose-inducible promoter (*P*_*xyl*_) in front of the *cdsA* gene, as described previously [10]. (C) PC micrographs of wild-type walled cells and L-forms (*P*_*spac*_-*ispA* and *P*_*spac*_-*hepS*) taken from the cultures shown in (B). The scale bar represents 5 μm. (D) Effects of transposon mutations on cell growth in the absence of a cell wall. *B*. *subtilis* strains, BS115 (Wt; *P*_*xyl*_-*murE*), LR2 (*ispA*; *P*_*xyl*_-*murE ispA^∗^*), YK1816 (*ndh*; *P*_*xyl*_-*murE ndh*::*tn*), YK1817 (*qoxB*; *P*_*xyl*_-*murE qoxB*::*tn*), YK1818 (*ctaB*; *P*_*xyl*_-*murE ctaB*::*tn*), and YK1522 (*mhqR*; *P*_*xyl*_-*murE mhqR*::*tn*) were grown on NA and MSM plates with (MurE ON) and without (MurE OFF) 2% xylose at 30°C for 2 days (MurE ON) and 3 days (MurE OFF). (E) PC micrographs of L-forms taken from the cultures shown in (D). The scale bar represents 5 μm.

IspA catalyzes the formation of farnesyl pyrophosphate (FPP) in the polyprenoid synthetic pathway [9] (Figure 2A). This pathway leads to the formation of two lipid molecules: heptaprenyl diphosphate (HPP), required for synthesis of menaquinone (MQ), which is involved in the electron transport chain (ETC) system, and undecaprenyl pyrophosphate (UPP), required for synthesis of the precursors for peptidoglycan (lipid II) and wall teichoic acid. If the *ispA* mutation works through one of these pathways, repression of either *hepS* (encoding HPP synthase) or *uppS* (encoding UPP synthase) should also promote L-form growth. Figure 2B shows a comparison of the effects of repression (isopropyl β-D-1-thiogalactopyranoside (IPTG)-dependent *P*_*spac*_ constructs) of *hepS*, *uppS*, or *ispA* on growth of walled cells or L-forms. When grown as walled cells, all three strains grew similarly to the wild-type in the presence of IPTG (left plate). In the absence of IPTG (center plate) neither the *hepS* nor the *uppS* repression strain grew, consistent with the essential roles for UppS and HepS in walled cells [11]. Repression of *ispA* did not suppress growth, presumably because it has a paralog, *hepT*, which encodes a component of HPP synthase [11]. We then induced L-form growth by inhibiting the PG precursor pathway with D-cycloserine (DCS), which inhibits the D-alanine-D-alanine ligase, Ddl (see [3]) (the FtsZ-targeting antibiotic 8j was added to kill walled cells; [7]). As expected, repression of *ispA* enabled growth on the L-form selective plates (right-hand panel of Figure 2B). Repression of *hepS* also supported growth on L-form plates, but *uppS* did not. Phase contrast microscopy confirmed the presence of heterogenous spheroidal cells on the *hepS* repression plate, similar to the L-forms induced by *ispA* repression (Figure 2C). These results suggest that the effect of the *ispA* mutation on L-form growth operates through the HPP/MQ pathway rather than the lipid II pathway.

In previous experiments selecting de novo L-form variants, we repeatedly found that the most robust colonies tended to have mutations in the *ispA* gene [5, 7]. To find out whether mutations in genes other than *ispA* would support L-form growth, we performed a screen in cells containing a second copy of *ispA*. A transposon mutant library was made in cells containing *P*_*xyl*_*-murE* (class 1 mutation) grown in the walled state (presence of xylose). L-forms were selected from the mutant library on plates containing sucrose as an osmoprotectant (but no xylose) and the cell division inhibitor, 8j. Six independent transposon mutants were selected, checked by backcrossing, and the sites of transposon insertion were determined by sequencing. Two mutations lay in each of the *ndh* and *qoxB* genes, and single insertions were found in *ctaB* and *mhqR* (Figures 2D and 2E). As shown in Figure 2A, three of these genes encode products involved in the ETC system: *ndh* encodes a major NADH dehydrogenease [12]; *qoxB* encodes cytochrome *aa*_*3*_ quinol oxidase subunit I [13]; *ctaB* encodes heme O synthase [14]; and *mhqR* encodes a transcriptional repressor for genes induced by the thiol-specific oxidative and/or electrophile stress response [15]. Figures 2D and 2E show the enabling effects of these mutations on L-form growth. Taken collectively, these results suggest that L-form growth, under the conditions we normally use, requires a reduction of ETC activity, and the identification of an *mhqR* mutation further suggests that reactive oxygen species (ROS) originating from the ETC pathway prevent L-form growth. For reasons that are not yet clear, L-forms generated by either *ispA* or *mhqR* mutations were able to grow in liquid medium, whereas the others would only grow on solid agar plates (data not shown).

### Abnormally Increased Cellular ROS Levels in Protoplasts Are Reduced by Switching into the L-Form State

**Figure 3:**
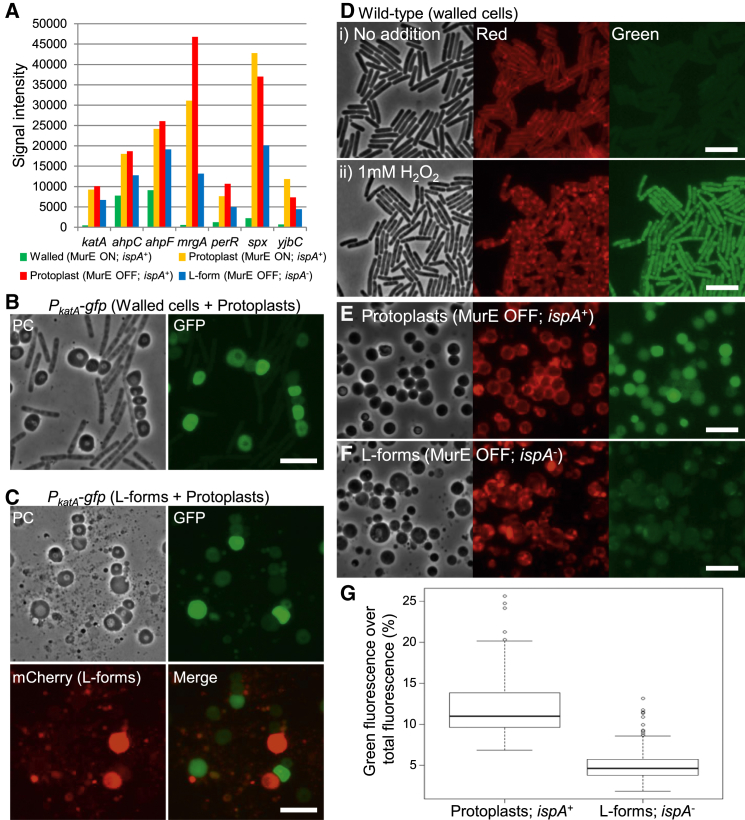
Increased ROS Production in Protoplasts and Its Suppression by an *ispA* Mutation (A) Expression patterns of several PerR regulated genes cultured in the walled (green; strain BS115; *P*_*xyl*_-*murE*, 2% xylose), protoplast (yellow and red; strain BS115; *P*_*xyl*_-*murE*, 2% or no xylose), or L-form (blue; strain LR2; *P*_*xyl*_-*murE ispA^∗^*, no xylose) states. See also Figure S1A and Table S1. (B) PC micrograph of a mixture of exponentially growing *B*. *subtilis* walled cells (YK2003; *P*_*xyl*_-*murE amyE*::*P*_*katA*_-*gfp*, 2% xylose) and an overnight culture of protoplasts (YK2003; no xylose) in NB and MSM (PC). The right panel shows the corresponding GFP image. The scale bar represents 5 μm. (C) Effect of *ispA* mutation on *P*_*katA*_ activity in protoplasts. PC micrograph of a mixture of overnight cultures of protoplasts (YK2003; *P*_*xyl*_-*murE amyE*::*P*_*katA*_-*gfp*, no xylose) and L-forms (YK2005; *P*_*xyl*_-*murE ispA^∗^ amyE*::*P*_*katA*_-*gfp aprE*::*P*_*rpsD*_-*mcherry*, no xylose) in NB and MSM (PC). The corresponding epifluorescence images of GFP and mCherry and the merge of GFP and mCherry are shown, respectively. The scale bar represents 5 μm. (D–F) The fluorescent probe C_11_-BODIPY^581/591^ was used as an indicator of lipid peroxidation. The probe undergoes a shift from red to green fluorescence emission upon peroxidation (see Supplemental Experimental Procedures). The scale bar represents 5 μm. (D) PC and corresponding epifluorescence micrographs (red and green fluorescence channels) of *B*. *subtilis* wild-type strain 168CA, grown in NB and MSM with (ii) and without (i) 1 mM H_2_O_2_. (E and F) PC and corresponding epifluorescence micrographs (red and green fluorescence channels) of overnight cultures of protoplasts (BS115; *P*_*xyl*_-*murE*, E) or L-forms (LR2; *P*_*xyl*_-*murE ispA*^∗^, F) in NB and MSM without xylose. (G) The relative signal intensity of green fluorescence over total fluorescence (green + red fluorescence) of protoplasts (left; *ispA*^+^ [n = 201]) and L-forms (right; *ispA*^−^ [n = 199]). The signal intensity was obtained from similar images to (E) and (F) by ImageJ. Boxplots represent median (horizontal black lines), the upper and lower quartile values (boxes), and the most extreme data points within 1.5 times interquartile ranges (whiskers). The tendency of the weakness on the green fluorescent intensity in the L-forms is statistically significant (Student’s t test, p < 0.01). Student’s t test and the preparation of a boxplot were performed by the statistics software package, R.

In general, all aerobic organisms use oxygen as the terminal electron acceptor for efficient energy production. However, ROS is also generated as a by-product through the metabolism of molecular oxygen, and this causes damage to nucleotides, proteins, and lipids ‎ [16, 17]. The cell therefore has various genetic systems to respond to oxidative stress. To investigate whether the oxidative stress response was induced in protoplasts, we compared the gene expression patterns of protoplasts (*P*_*xyl*_-*murE*, with and without xylose) to those of walled cells (*P*_*xyl*_-*murE*, with xylose) and L-forms (*P*_*xyl*_-*murE ispA^∗^*, without xylose) using microarrays. The results showed that the transcription of 103 genes was specifically induced in protoplasts (Tables S1 and S2). Many of those genes (43 genes) have roles against various stresses, including resistance to oxidative and electrophile stress (13 genes), cell envelope stress (12 genes), and heat shock (6 genes). As shown in Figure 3A (see also Figure S1A), strong induction of genes belonging to the PerR regulon, which is induced by the peroxide-induced oxidative stress [18], was detected in protoplasts. In comparison, many essential genes or functions, such as DNA replication and protein synthesis, for the growth of normal walled state were downregulated in protoplasts (Table S1). The stringent response (Figure S1B), which is induced by amino acid starvation or other stresses [19], may be largely responsible for these downregulation effects. To confirm induction of the oxidative stress response in protoplasts, we examined expression of the *katA* promoter using a *P*_*katA*_-*gfp* fusion [20]. The *katA* gene encodes a vegetative catalase and is part of the PerR regulon. Figure 3B shows a mixture of exponentially growing wild-type walled cells and an overnight culture of protoplasts with PG precursor synthesis repressed (*P*_*xyl*_-*murE*, no xylose) in L-form medium. An obvious GFP signal was detected in most protoplasts, whereas little or no detectable GFP fluorescence was seen in the walled cells (Figure 3B, GFP). We then examined the *P*_*katA*_ activity in a mixture of the protoplasts (unlabelled) and L-forms (*P*_*xyl*_-*murE ispA^∗^ aprE*::*mCherry*, red cells expressing mCherry). Whereas strong GFP signals were present in many protoplasts, the GFP signals in L-forms were much weaker (Figure 3C). The result is consistent with oxidative stress being significantly induced in protoplasts, but not in L-forms.

To assay more directly for ROS production in protoplasts, we took advantage of a fluorescent fatty acid analog, C_11_-BODIPY^581/591^, which has been used as an indicator of oxidative damage to lipids, i.e., lipid peroxidation [21, 22]. The probe is incorporated into membranes, and the fluorescent properties in the red range of the visible spectrum (emission maximum 595 nm) in fluorescence microscopy shift to the green range (520 nm) upon free radical-induced oxidation. In a control experiment with wild-type *B*. *subtilis* walled cells exponentially growing in sucrose-based L-form medium, the typical rod-shaped cells exhibited a highly regular and smooth red fluorescence at the cell surface, but no clear green fluorescence was detectable (Figure 3D, i). After treatment with H_2_O_2_, green fluorescence was evident within most cells, and the staining was patchy and irregular (Figure 3D, ii). In contrast to the walled cells, green fluorescence was readily detectable in protoplasts (*P*_*xyl*_-*murE*, no xylose) in the absence of exogenous oxidant (Figure 3E), suggesting lipid peroxidation by endogenous oxygen radicals. Importantly, the fluorescent shift was largely suppressed in L-forms carrying an *ispA* mutation (*P*_*xyl*_-*murE ispA^∗^*, no xylose) (Figures 3F and 3G). Thus, the cellular ROS levels are indeed increased in protoplasts, but they are suppressed by reduction of the ETC activity via *ispA* mutation. Consistent with the idea that the increased cellular ROS levels in protoplasts originate from the ETC pathway, strong induction of various genes involved in the tricarboxylic acid (TCA) cycle was detected in protoplasts by microarray experiments (Figures S1C and S1D).

### Oxidative Stress Response Genes Are Required to Support L-Form Growth

**Figure 4:**
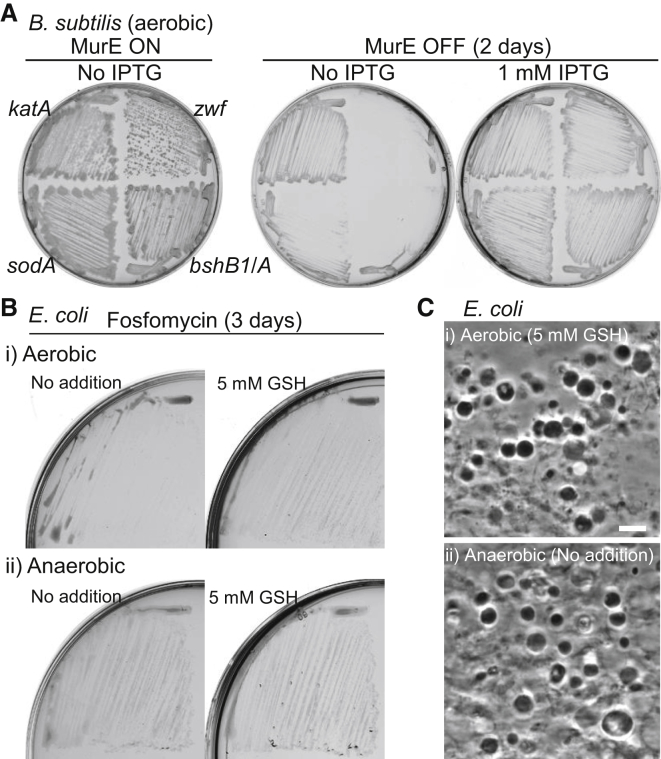
Effects of ROS on L-Form Growth (A) Effect of repression of antioxidant systems on *B*. *subtilis* L-form growth in aerobic conditions. *B*. *subtilis* strains with the following mutations were cultured on NA and MSM plates with (MurE ON) or without (MurE OFF) 2% xylose at 30°C for 2 days in the presence or absence of 1 mM IPTG: *P*_*xyl*_-*murE ispA*^∗^ with *P*_*spac*_-*katA* (YK2027), *P*_*spac*_-*sodA* (YK2028), *P*_*spac*_-*bshB1*/*A* (YK1604), and *P*_*spac*_-*zwf* (YK1584). (B) *E*. *coli* L-form growth on L-form plates (NB and MSM 1% agar with 400 μg/ml fosfomycin) at 30°C for 3 days. Growth of the *E*. *coli* strain RM345 (*ΔmurA* containing the unstable plasmid pOU82-*murA* [3] on L-form plates with or without 5 mM reduced GSH in aerobic (i) or anaerobic (ii) conditions. See also Figure S2. (C) PC micrograph of *E*. *coli* L-forms taken from the cultures shown in (B). The scale bar represents 5 μm.

As described above, microarray experiments showed specific induction of genes in the PerR regulon and other genes for resistance against oxidative stress in protoplasts (Figure S1A). However, in many cases, the expression levels in L-forms were also significantly higher than those of walled cells. We wondered whether the stress response genes might be needed to protect cells against oxidative stress during L-form growth. To test this, we inserted an IPTG-dependent promoter in front of four genes or operons encoding antioxidant systems, *katA* (main vegetative catalase) [23], *sodA* (superoxide dismutase) [24], *bshB1*/*bshA* (bacillithiol synthesis) [25], and *zwf* (glucose 6-phosphate dehydrogenase; for NADPH generation) [26], in strain LR2 (*P*_*xyl*_-*murE ispA^∗^*). Note that the thioredoxin system is essential for viability in walled cells and that *B*. *subtilis* lacks a glutathione (GSH) system. In the walled state, none of the mutations had a significant effect on cell growth (Figure 4A, MurE ON/No IPTG). However, in L-form state (MurE OFF/No IPTG), repression of the *sodA*, *bshB1* operon, or *zwf* genes severely inhibited growth. Repression of *katA* did not abolish L-form growth, but this was not completely surprising as *B. subtilis* has several paralogous genes and an alkyl hydroperoxide reductase [27]. Induction of the antioxidant systems by addition of IPTG restored growth in each case (Figure 4A, MurE OFF/1 mM IPTG). Thus, several antioxidant systems seem to be crucial for L-form proliferation in *B*. *subtilis*.

### Reduction of ROS Promotes L-Form Growth in *E*. *coli*

We have previously reported that L-forms of the Gram-negative bacterium *E. coli* do not require an *ispA*-like mutation for proliferation on L-form plates (containing sucrose as osmoprotectant and fosfomycin, an inhibitor of the PG precursor pathway) [3]. Nevertheless, since the growth of *E*. *coli* L-forms is apparently much slower than that of *B*. *subtilis*, we wondered whether this was again due to oxidative damage. If so, then treatment of cells with a ROS scavenger such as reduced GSH might improve the growth of *E*. *coli* L-forms. *E*. *coli* walled cells were streaked on L-form selective plates with and without GSH (Figure 4B, i). In the absence of GSH, discrete L-form colonies were barely visible after 3 days of incubation, though a lawn of visible colonies emerged after about 5 days (Figures S2A and S2B). In contrast, in the presence of GSH, significant L-form growth was seen within 3 days (Figure 4B, i; Figure 4C, i), supporting the idea that a reduction of cellular ROS levels promotes *E*. *coli* L-form growth. If the cellular ROS levels are increased through the metabolism of molecular oxygen in the ETC pathway in *E*. *coli* cell-wall-deficent cells, then anaerobic culture should also promote the growth of L-forms. Strikingly, under anaerobic conditions, significant *E*. *coli* L-form growth was seen within 3 days even in the absence of GSH (Figure 4B, ii; Figure 4C, ii). We also examined the effects of an *ispA* mutation on *B*. *subtilis* L-form growth under anaerobic condition and found that *ispA* muation was no longer required for L-form growth when oxygen was depleted (Figures S2C and S2D).

## Discussion

We have proposed that L-form proliferation may provide insights into an ancient mechanism used in primordial cells before the invention of the cell wall [3, 7, 28]. In this report, we have found that the cellular ROS levels are abnormally increased in cell-wall-deficient cells and that a reduction of cellular ROS levels by repression of the ETC activity, addition of a ROS scavenger or anaerobic culture, promotes the growth of wall-free L-forms in both Gram-positive and Gram-negative bacteria. Therefore, oxidative damage could be an important impediment to L-form growth in a wide range of bacteria.

Why should the L-form transition or growth in the absence of cell wall result in increased oxidative damage? Cell wall synthesis is probably a major drain on cellular resources under normal conditions, so a block in cell wall synthesis probably leads to major changes in cell metabolism. Uridine 5′-diphospho-*N*-acetyl-d-glucosamine (UDP-GlcNAc), which is an essential cell wall precursor for both lipid II and WTA synthesis (Figure 2A), is generated from fructose-6-phospate via central carbon metabolism, through the action of the *glmS*, *glmM*, and *gcaD* gene products. The *glmS* riboswitch is a ribozyme that self-cleaves upon binding glucosamine-6-phosphate, the product of the enzyme encoded by *glmS* [29]. Inhibition of PG synthesis would result in increased cellular levels of glucosamine-6-phosphate, and the subsequent repression of *glmS* could increase glycolytic flux due to a reduction in utilization of fructose-6-phospate for UDP-GlcNAc synthesis. This could, in turn, result in an increase of cellular pyruvate levels and stimulate flux into the TCA cycle. Consistent with this, our results suggest that the stimulation of ROS production in wall-deficient cells is most likely due to an increase in TCA cycle flux (see Figures S1C and S1D), leading to increased synthesis of NADH and FADH_2_, which are the major substrates for the ETC pathway. The subsequent stimulation of ETC flux results in an increase of ROS generation as a by-product of the metabolism of molecular oxygen. Importantly, Kohanski et al. [30] have proposed a model that bactericidal antibiotics, including cell wall antibiotics, work at least in part by stimulating ROS production through a burst of NADH comsumption by the ETC pathway, although this is currently controversial [31–33]. Nevertheless, our results suggest that physiological compensation for the metabolic imbalance that occurs when the normal large flux to cell wall synthesis is blocked is crucial for the proliferation of cell-wall-free L-form bacteria. Apart from the importance for understanding early forms of cellular life, the ability to grow without a cell wall in L-forms also provides new insights into the mode of action of antibiotics that target the bacterial cell wall.

## Experimental Procedures

Experimental Procedures are described in the Supplemental Information.

## Author Contributions

Y.K., R.M., and J.E. designed the experiments. Y.K., R.M., L.J.W., P.D.-C., and T.O. performed the experiments. Y.K., R.M., and T.O. analyzed the data. Y.K. and J.E. wrote the manuscript.

## Accession Numbers

The accession number for the microarray data reported in this paper is ArrayExpress: E-MTAB-3380.

## Supplemental Information

Document S1. Supplemental Experimental Procedures, Figures S1 and S2, and Table S1

Table S2. Genes Upregulated or Downregulated in Protoplasts, Related to Figure 3A

Movie S1. Effects of *ispA* Mutation in Protoplast Growth, Related to Figure 1B Time-lapse series with a microfluidic system showing the growth of protoplasts of strains BS115 (*P*_*xyl*_-*murE*) and 4738 (*P*_*xyl*_-*murE ispA^∗^ aprE*::*P*_*rpsD*_*-mcherry*) in NB and MSM (no xylose), from which the panels in Figure 1B were obtained. Phase contrast and the corresponding mCherry images were acquired automatically every 5 min.

Document S2. Article plus Supplemental Information
