# Supplementary material for: Cell Growth of Wall-Free L-Form Bacteria Is Limited by Oxidative Damage
Source: Curr Biol. 2015 Jun 15;25(12):1613–8. doi: 10.1016/j.cub.2015.04.031 (PMC4510147; doi:10.1016/j.cub.2015.04.031)
Supplement: Document S1. Supplemental Experimental Procedures, Figures S1 and S2, and Table S1 [file mmc1.pdf]

Current Biology

Supplemental Information

Cell Growth of Wall-Free L-Form Bacteria Is Limited by Oxidative Damage

Yoshikazu Kawai, Romain Mercier, Ling Juan Wu, Patricia Domínguez-Cuevas, Taku Oshima, and Jeff Errington

Figure S1

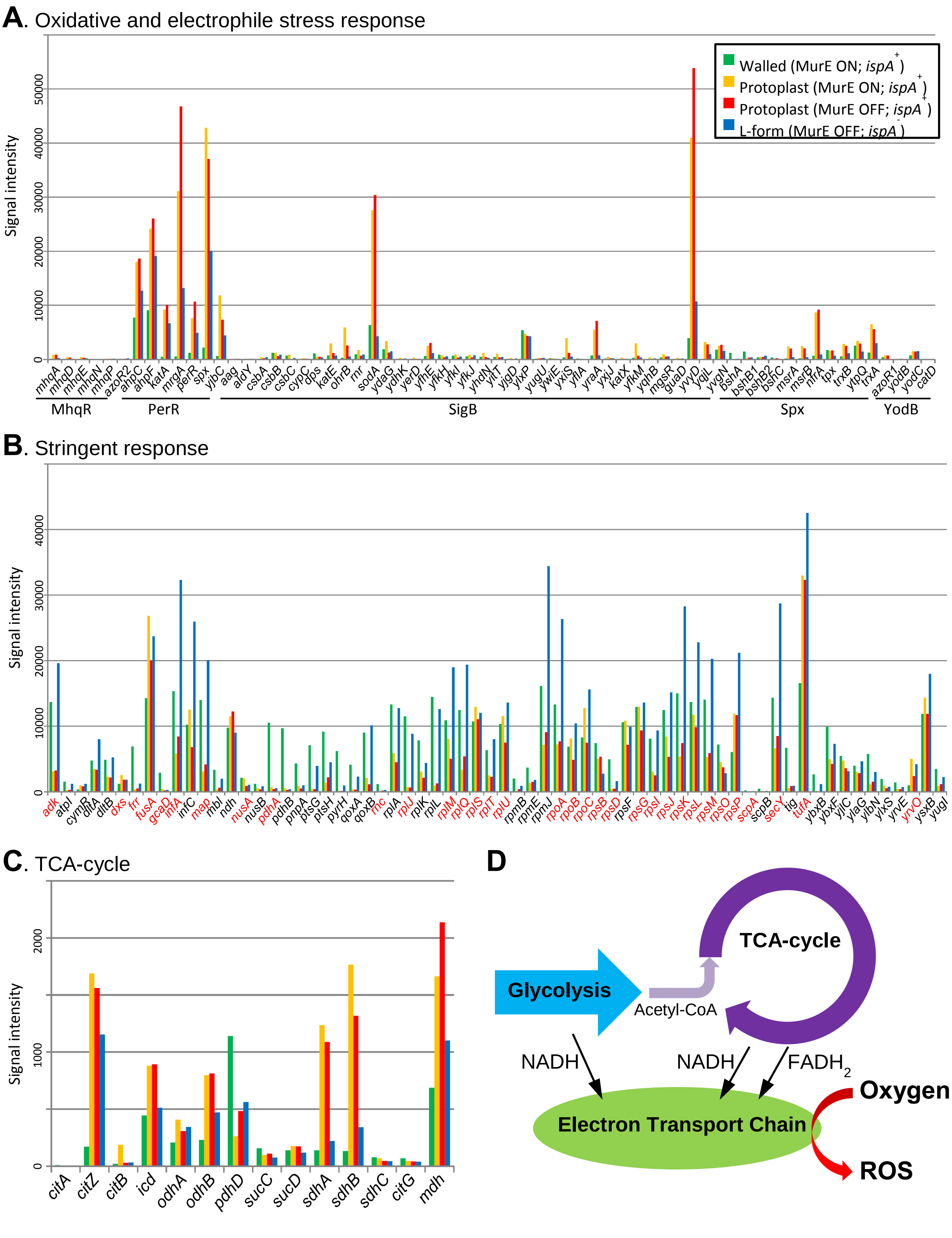

A. Oxidative and electrophile stress response
Signal intensity
Walled (MurE ON; ispA+)
Protoplast (MurE ON; ispA+)
Protoplast (MurE OFF; ispA+)
L-form (MurE OFF; ispA-)
MhqR
PerR
SigB
Spx
YodB
B. Stringent response
Signal intensity
C. TCA-cycle
Signal intensity
citA
citZ
citB
icd
odhA
odhB
pdhD
sucC
sucD
sdhA
sdhB
sdhC
citG
mdh
D
TCA-cycle
Glycolysis
Acetyl-CoA
NADH
NADH
FADH2
Electron Transport Chain
Oxygen
ROS

Figure S1, related to Figure 3. Expression patterns of various genes in protoplasts and L-forms

(A-C) Expression patterns of various genes in walled (green, strain BS115; P_{xyl}-*murE*, 2% xylose), protoplast (yellow, BS115; P_{xyl}-*murE*, 2% xylose, and red, P_{xyl}-*murE*, no xylose) or L-form (blue, LR2; P_{xyl}-*murE ispA**, no xylose) cells.

(A) Expression patterns of genes related to resistance against oxidative and electrophile stress. Their corresponding transcriptional regulators, MhqR, PerR, SigB, Spx and YodB, are shown below the genes.

(B) Expression patterns of genes affected by the stringent response. Genes essential for viability of normal walled cells are indicated in red.

(C) Expression patterns of genes in the TCA cycle.

(D) Schematic representation of the links between the TCA cycle, the ETC pathway and ROS generation. See detail in Discussion.

Figure S2

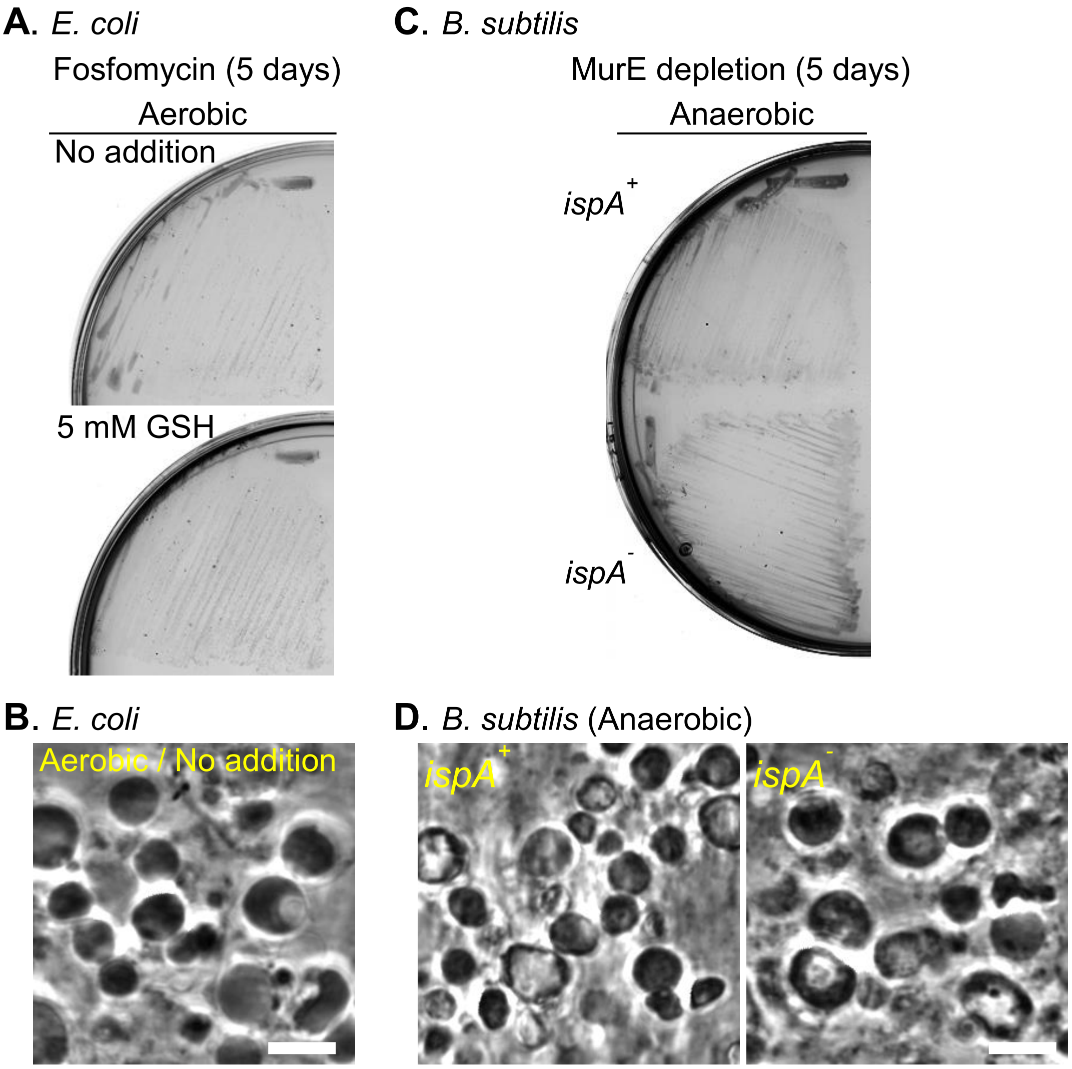
A. E. coli
Fosfomycin (5 days)
Aerobic
No addition
5 mM GSH
C. B. subtilis
MurE depletion (5 days)
Anaerobic
ispA+
ispA-
B. E. coli
Aerobic / No addition
D. B. subtilis (Anaerobic)
ispA+
ispA-

Figure S2, related to Figure 4. Effects of ROS on L-form growth

(A) Growth of the *E*. *coli* strain RM345 (*ΔmurA* and containing the unstable plasmid pOU82-*murA* [S1] on L-form plates (NB/MSM 1% agar with 400 μg/ml fosfomycin) with or without 5 mM reduced glutathione (GSH) at 30°C for 5 days under aerobic conditions.

(B) Phase contrast micrograph of *E*. *coli* L-forms taken from the culture shown in panel A (No GSH addition). Scale bar represents 5 μm.

(C) Growth of the *B*. *subtilis* strains BS115 (P_{xyl}-*murE*) and LR2 (P_{xyl}-*murE ispA**) on L-form plates (no xylose) with 10 mM Nitrate at 30°C for 5 days under anaerobic conditions. Note that *B. subtilis* was unable to grow in the absence of molecular oxygen as a terminal electron acceptor [S2].

(D) Phase contrast micrograph of *B*. *subtilis* L-forms taken from the cells shown in panel C under aerobic situation. Scale bar represents 5 μm.

Table S1. Summary of the microarray results

Function categories	P > L[a]		L > P[b]	
	P > W and L[c] (P-3hr > W)[d]	P ≒ W > L[e]	L > W and P[f]	L ≒ W > P[g]
**Carbon metabolism**	11 (7)	4	19	6
**Amino acid metabolism**	5 (5)	0	1	16
**Nucleotide metabolism**	1 (1)	2	0	4
**Lipid metabolism**	1 (0)	1	0	3
**Electron transport / ATP synthesis**	4 (1)	0	3	12
**Metal Homeostasis**	5 (4)	0	0	5
**DNA replication / segregation / repair**	1 (1)	0	0	6
**RNA and protein synthesis**	1 (1)	1	3	23
**Cell wall synthesis / cell division**	2 (1)	1	3	4
**Stress response**	43 (26)	10	16	9
**Prophages**	3 (3)	1	38	2
**Unknown function**	20 (16)	12	11	28
**Others**	6 (4)	14	1	5
**Total (number of genes)**	**103 (70)**	**46**	**95**	**123**

Total RNA was isolated from *B. subtilis* cells (OD_{600nm}=0.2~0.3) cultured in NB/MSM at 30°C (see detail in Experimental Procedures). P; overnight culture (18hr incubation) of protoplasts of strain BS115 (P_{xyl}-*murE*, no xylose), L; exponentially growing L-forms of strain LR2 (P_{xyl}-*murE ispA**, no xylose), W; exponentially growing walled cells of strain BS115 (P_{xyl}-*murE*, 2% xylose) and P-3hr; protoplasts of strain BS115 (P_{xyl}-*murE*, no xylose) incubated for 3 hr after conversion to protoplasts.

[a]Number of genes that revealed higher expression levels (more than three times) in P than L.

[b]Number of genes that revealed higher expression levels (more than three times) in L than P.

[C]Number of genes that revealed higher expression levels (more than three times) in P than W and L. (see detail in Table S2i)

[d]Number of genes that revealed higher expression levels (more than three times) in P-3hr than W.

[e]Number of genes that revealed higher expression levels (more than three times) in P than L, and no significant difference between P and W. (see detail in Table S2ii)

[f]Number of genes that revealed higher expression levels (more than three times) in L than W and P. (see detail in Table S2iii)

[g]Number of genes that revealed higher expression levels (more than three times) in L than P, and no significant difference between L and W. (see detail in Table S2iv)

Bacterial strains and plasmid used in this study

**Strain**	**Relevant genotype**	**Reference**
B. subtilis		
168CA	*trpC2*	Lab. stock
BS115	168CA Ω*spoVD*::*cat* P_{xyl}-*murE* Ω*amyE*::*xylR tet*	Lever et al., 2009 [S3]
LR2	BS115 *xseB** (Frameshift 22T>-)[a]	Mercier et al., 2013 [S4]
SH517	**amyE**::P_{katA}**-gfp spc trpC2 pheA1**	Hoover et al., 2010 [S5]
Bss307	**168CA aprE::**P_{rpsD}**-mcherry spc**	S. Syvertsson, unpublished
4738	**LR2 aprE::**P_{rpsD}**-mcherry spc**	This study
RM82	**LR2 amyE::**P_{xseB}**-xseB-ispA spc**	Mercier et al., 2013 [S4]
YK1424	168CA Ω*ispA*::pMutin4-*erm*-P_{spac}-*ispA*	Mercier et al., 2013 [S4]
YK1494	BS115 **amyE::**P_{xseB}**-xseB-ispA spc**	This study
YK1450	168CA Ω*hepS*::pMutin4-*erm*-P_{spac}-*hepS*	This study
YK1522	BS115 *mhqR*::*TnYLB-1* (*kan*)	This study
YK1584	LR2 Ω*zwf*::pMutin4-*erm*-P_{spac}-*zwf*	This study
YK1604	LR2 Ω*mgsA*::pMutin4-*erm*-P_{spac}-*bshB1*	This study
YK1694	168CA *xseB*::*TnYLB-1* (*kan*)[b] *amyE*::P_{xyl}-*accDA spc*	Mercier et al., 2013 [S4]
YK1816	BS115 *ndh*::*TnYLB-1* (*kan*)	This study
YK1817	BS115 *qoxB*::*TnYLB-1* (*kan*)	This study
YK1818	BS115 *ctaB*::*TnYLB-1* (*kan*)	This study
YK1889	168CA Δ*uppS*::*kan* pLOSS-P_{spac}-*uppS-erm* ΩP_{xyl}-*cdsA spc*	Kawai et al., 2014 [S6]
YK2003	**BS115 amyE**::P_{katA}**-gfp spc**	This study
YK2005	LR2 *aprE*:: **P_{rpsD}**-*mCherry kan amyE*::P_{katA}-*gfp spc*	This study
YK2027	LR2 Ω*katA*::pMutin4-*erm*-P_{spac}-*katA*	This study
YK2028	LR2 Ω*sodA*::pMutin4-*erm*-P_{spac}-*sodA*	This study
E. coli		
TB28	MG1655 **ΔlacIZYA**	Lab. stock
RM345	TB28 **ΔmurA::kan** pOU82-**murA**	Mercier et al., 2014 [S1]
**Plasmid**		
pMutin4	*bla erm* P_{spac} *mcs lacZ lacI*	Vagner et al., 1998 [S7]
pM4-P_{spac}-*hepS*	*bla erm* P_{spac}-*hepS*-5' *lacZ lacI*	This study
pM4-P_{spac}-*zwf*	*bla erm* P_{spac}-*zwf*-5' *lacZ lacI*	This study
pM4-P_{spac}-*bshB1*	*bla erm* P_{spac}-*bshB1*-5' *lacZ lacI*	This study
pM4-P_{spac}-*katA*	*bla erm* P_{spac}-*katA*-5' *lacZ lacI*	This study
pM4-P_{spac}-*sodA*	*bla erm* P_{spac}-*sodA*-5' *lacZ lacI*	This study

cat, chloramphenicol; *tet*, tetracyclin; *erm*, erythromycin; *spc*, specctinomycin; *kan*, kanamycin; *bla*, β-lactamase; represent resistant genes, respectively

[a & b] These mutations repress expression of the *ispA* gene [S4]

Primers

Primer	nucleotide sequence
pM4-P_{spac}-*hepS*-F	GAA**GAATTC**TGTGAATTTGGGGACAAG
pM4-P_{spac}-*hepS*-R	GGA**GGATCC**TGGCGGTTTTTGTTTTCG
pM4-P_{spac}-*zwf*-F	GGG**GAATTC**GGTGTACTAAAATAAAGCTTCG
pM4-P_{spac}-*zwf*-R	GGG**GGATCC**ACGTCAAACGGGTGATAGTAA
pM4-P_{spac}-*bshB1*-F	GAA**GAATTC**TTGATCGCGCATGACAAG
pM4-P_{spac}-*bshB1*-R	GGA**GGATCC**AATGACAAGATCGAGTGC
pM4-P_{spac}-*katA*-F	GAA**GAATTC**CCAAGAGGTGATAACATGAG
pM4-P_{spac}-*katA*-R	GGA**GGATCC**CGGCAACTGTTGAGAAACG
pM4-P_{spac}-*sodA*-F	GAA**GAATTC**CTAAGGAGGAATTATCATGGC
pM4-P_{spac}-*sodA*-R	GGA**GGATCC**TTTGTGGTTCGCGTGTCCG

Supplemental Experimental Procedures

Bacterial strains, plasmids, primers and growth conditions

The bacterial strains, plasmid constructs and primers for PCR analysis in this study are shown in Supplemental Information. DNA manipulations were carried out using standard methods. *B. subtilis* and *E. coli* walled cells were grown on nutrient agar (NA, Oxoid) or in nutrient broth (NB, Oxid) at 30°C. *B. subtilis* L-forms and protoplasts were grown in osmoprotective medium composed of 2 x magnesium-sucrose-maleic acid (MSM) pH7 (40 mM $MgCl_2$, 1 M sucrose, and 40 mM maleic acid) mixed 1:1 with 2 x NB or 2 x NA at 30°C. *B. subtilis* L-forms and protoplasts were cultured in liquid medium without shaking. *E. coli* L-forms were grown on osmoprotective medium composed of 2 x MSM mixed NB with 2% agar at 30°C. Anaerobic growth condition was maintained using anaerobic atmosphere generation bags (AnaeroGen^{TM}, Oxid) in an anaerobic jar for growth on plates. Supplements, 1 or 0.5 mM IPTG, 1 or 2% xylose, 5 mM glutathione (Sigma-Aldrich) were added when indicated. When necessary, antibiotics were added to media at the following concentrations: 100 µg/ml ampicillin, 1 µg/ml erythromycin, 5 µg/ml kanamycin, 50 µg/ml spectinomycin, 300 µg/ml, 400 µg/ml D-cycloserine, 400 µg/ml fosfomycin, 200 µg/ml PenG and/or 1 µg/ml 8J (FtsZ inhibitor, [S8]). Experimental Procedures were also described in Supplemental Information.

Construction of IPTG-inducible mutants

The first 200~300 bp of the *hepS*, *zwf*, *bshB1*, *katA* or *sodA* gene containing Shine–Dalgarno sequence was amplified by PCR from genomic DNA of the wild-type strain 168CA using the primers in above table, then cloned between the EcoRI and BamHI sites of plasmid pMutin4 [S7], creating pM4-P_{spac}-*hepS*, *zwf*, *bshB1*, *katA* or *sodA*, respectively (see starin table). The resulting plasmids were introduced into *B. subtilis* to generate YK1450, YK1584, YK1604, YK2027 and YK2028, respectively (strain table). In those strains, the full-

length *hepS*, *zwf*, *bshB1*, *katA* or *sodA* gene is expressed from the IPTG-inducible promoter P_{spac}.

Transposon mutagenesis

Transposon mutagenesis was performed essentially as described previously [S9, S10]. To screen for mutants that restored of viability of strain YK1494 (P_{xyl}-*murE amyE*::*ispA*), containing a second copy of *ispA* gene at the *amyE* locus, in the absence of xylose on NA/MSM plates, cells were transformed with the transposon plasmid pMarB and transformants were selected on NA containing 1% xylose at 30°C. Several transformants were picked and individually grown for 8 h in NB with 1% xylose at 30°C. The cells from each culture were then plated and incubated overnight at 50°C on NA plates containing kanamycin and 1% xylose, erythromycin and 1% xylose. We then selected the plate that gave the highest ratio of kanamycin-resistant colonies versus erythromycin-resistant colonies. The selected culture was used to generate a library of about 100,000 colonies on NA containing kanamycin and 1% xylose at 50°C. Mutants that restored the viability to strain YK1494 in the absence of xylose were selected from the library on NA/MSM plates (no xylose) containing 1 μg/ml 8j. Genomic DNA of the mutants were isolated and backcrossed into strain BS115 (P_{xyl}-*murE*) to confirm that the restoration of growth was not due to a second site mutation. Mutants that had stable suppressor mutations linked to a transposon insertion were confirmed by back crossing and were subjected to inverse PCR amplification and sequencing of the transposon insertion site as described previously [S9]. We isolated six independent transposon insertions in *ctaB* or *mhqR*, or two distinct positions of *ndh* or *qoxB*.

Protoplast and L-form preparation in Liquid medium

Exponentially growing *B*. *subtilis* walled cells (OD_{600nm} of 0.2~0.3) in NB/MSM medium with appropriate supplements were harvested and resuspended in fresh NB/MSM containing lysozyme (100 μg/ml) and supplements, if required. The cells were incubated at 37°C with shaking for 1 hr. For protoplast growth (L-form transition), the protoplasts were diluted

(1/1000) into fresh NB/MSM containing supplements, if required, and incubated at 30°C without shaking, as described previously [S4].

RNA isolation

Isolation of total RNA of *B. subtilis* cultures (OD_{600nm} of 0.2~0.3) was carried out as described previously [S11]. Strain BS115 (P_{xyl}-*murE*) was cultured in 10 ml NB/MSM medium with 2% xylose (walled cells). The cells were harvested and resuspended in fresh NB/MSM containing lysozyme (100 µg/ml) with and without 2% xylose. The cell cultures were incubated at 37°C with shaking for 1 hr to generate protoplasts. The protoplast cultures were further incubated in NB/MSM containing 8j and PenG with or without 2% xylose at 30°C for 3 hr or 18 hr. L-forms were prepared by using protoplast of strain LR2 (P_{xyl}-*murE ispA**), as described above.

Microarray analysis

The cDNA synthesis using 5 µg of purified total RNA and the terminal labeling of fragmented cDNAs were performed as described previously [S12]. Hybridization of Genechip *B. subtilis* Array with the labelled cDNA fragments and the scanning of the hybridized Genechip were performed with an appropriate hybridization condition and the program for the array, ProkGE-WS2, according to the manufacture's instruction (Affymetrix). The signal intensities of all probes on Genechip in each hybridization were normalized using the scaling program in GCOS software with a target signal intensity of 500, according to the manufacture's instruction (Affymetrix). On Affymetrix *B. subtilis* array, there are probes corresponding to intergenic regions, gene coding regions (for some genes, there are multiple gene sets) and control probes, then, we used the normarized intensities for the gene coding regions in this analysis. To summarise microarray results in Table 1, we selected the probes corresponding to the genes with adequate signal intensities [sum of the signal intensities of walled cells (P_{xyl}-*murE*, 2% xylose), Protoplasts (P_{xyl}-*murE*, no xylose) of 3 hr and 18 hr incubations, L-

forms (P_{xyl}-*murE ispA**, no xylose) > 400] to eliminate lower expression genes. ArrayExpress accession number is E-MTAB-3380.

Microfluidic system

Microfluidic experiments were carried out using a device described previously [S13]. Briefly, 3 ul of concentrated L-form (or protoplast) culture was added onto the cover glass which formed the bottom of the sample chamber, then a patterned agarose pad was placed (patterned side down) onto the culture in the sample chamber, trapping bacterial cells in the tracks of the agarose pad. The top of the chamber was then sealed with a plasma-treated cover glass (Agar Acientific Ltd, L46s20-5, coverglass 20x20 mm No.5). The assembly was left at room temperature or at 30°C for 20 min to allow plasma bond to set. Patterned agarose pads, with tracks of 1.5 um deep, were cast using an 'Intermediate PDMS mould' with 4% low melting point agarose (SeaPlague GTG Agarose from Lonsza, gelling temp 26–30°C) in L-form medium (NB/MSM/PenG), that set slowly at 30°C for 1 – 2h. The tracks were the repeat of a set of 3 tracks of 0.8 um, 0.9 um and 1.0 um wide, grouped into 15um x 20um blocks divided by gutters. Sample chambers were created by plasma-bonding a PDMS chamber block to a long cover glass (Agar Acientific Ltd, L4239-2, Coverglass 35x64 mm No.1.5). The PDMS chamber block also contained two buffer reservoirs on either side of, and connected to, the sample chamber, one for imputing fresh medium and the other as the outlet of the spent medium and bacterial cells that were not confined in the tracks. Growth medium was supplied continuously through the inlet reservoir from a 50 ml syringe, controlled by a syringe pump at a speed of 3 ml/h using the WinPump Term software (New Era).

Microscopy was performed on a Nikon Eclipse Ti inverted fluorescence microscope system fitted with an Apo TIRF objective (Nikon 60x/1.49 Oil). Light was transmitted from a 300 Watt xenon arc-lamp through a liquid light guide (Sutter Instruments) and images were collected using a HQ2-coolsnap camera (MAG Biosystems). The focus was maintained throughout the experiment using the Perfect Focus System (Nikon). All filters were Modified

Magnetron ET Sets from Chroma. The filter for GFP was 49002 ET-EGFP (FITC/CY2) (exciter ET470/40x; dichroicT495LP; emitter ET525/50M). For mCherry the filter used was 49005 ET-DsRed (TRITC/Cy3) (exciter ET545/30x; dichroicT570LP; emitter ET620/60m). Digital images were acquired and analysed using Frap-AI 7.7.5.0 (MAG Biosystems).

Lipid peroxidation

Lipid oxidation was detected using a fluorescent probe (C_{11}-BODIPY$^{581/591}$; Molecular Probes) as described previously [S14-S16] . Exponentially growing *B*. *subtilis* walled cells were cultured in NB/MSM with or without 1 mM H_2O_2 at 37°C for 1 hr, and then 5 μM C_{11}-BODIPY$^{581/591}$ was added to the culture and incubated for 1 hr at 30°C. The cells were used for microscopic analysis. For protoplasts and L-forms, 5 μM C_{11}-BODIPY$^{581/591}$ was added to overnight cultures and the cultures were incubated for 1 hr at 30°C before being used for microscopic analysis.

References

S1. Mercier, R., Kawai, Y., and Errington, J. (2014). General principles for the formation and proliferation of a wall-free (L-form) state in bacteria. eLife *3*.

S2. Nakano, M.M., and Zuber, P. (1998). Anaerobic growth of a “strict aerobe” (*Bacillus subtilis*). Annu. Rev. Microbiol. *52*, 165-190.

S3. Leaver, M., Dominguez-Cuevas, P., Coxhead, J.M., Daniel, R.A., and Errington, J. (2009). Life without a wall or division machine in *Bacillus subtilis*. Nature *457*, 849-853.

S4. Mercier, R., Kawai, Y., and Errington, J. (2013). Excess membrane synthesis drives a primitive mode of cell proliferation. Cell *152*, 997-1007.

S5. Hoover, S.E., Xu, W., Xiao, W., and Burkholder, W.F. (2010). Changes in DnaA-dependent gene expression contribute to the transcriptional and developmental

response of *Bacillus subtilis* to manganese limitation in Luria-Bertani medium. J. Bacteriol. *192*, 3915-3924.

S6. Kawai, Y., Mercier, R., and Errington, J. (2014). Bacterial cell morphogenesis does not require a preexisting template structure. Curr. Biol. *24*, 863-867.

S7. Vagner, V., Dervyn, E., and Ehrlich, S.D. (1998). A vector for systematic gene inactivation in *Bacillus subtilis*. Microbiol. *144*, 3097-3104.

S8. Adams, D.W., Wu, L.J., Czaplewski, L.G., and Errington, J. (2011). Multiple effects of benzamide antibiotics on FtsZ function. Mol. Microbiol. *80*, 68-84.

S9. Le Breton, Y., Mohapatra, N.P., and Haldenwang, W.G. (2006). In vivo random mutagenesis of *Bacillus subtilis* by use of TnYLB-1, a mariner-based transposon. Appl. Environ. Microbiol. *72*, 327-333.

S10. Kawai, Y., Daniel, R.A., and Errington, J. (2009). Regulation of cell wall morphogenesis in *Bacillus subtilis* by recruitment of PBP1 to the MreB helix. Mol. Microbiol. *71*, 1131-1144.

S11. Dominguez-Cuevas, P., Mercier, R., Leaver, M., Kawai, Y., and Errington, J. (2012). The rod to L-form transition of *Bacillus subtilis* is limited by a requirement for the protoplast to escape from the cell wall sacculus. Mol Microbiol *83*, 52-66.

S12. Kusuya, Y., Kurokawa, K., Ishikawa, S., Ogasawara, N., and Oshima, T. (2011). Transcription factor GreA contributes to resolving promoter-proximal pausing of RNA polymerase in *Bacillus subtilis* cells. J. Bacteriol. *193*, 3090-3099.

S13. Moffitt, J.R., Lee, J.B., and Cluzel, P. (2012). The single-cell chemostat: an agarose-based, microfluidic device for high-throughput, single-cell studies of bacteria and bacterial communities. Lab on a chip *12*, 1487-1494.

S14. Drummen, G.P., van Liebergen, L.C., Op den Kamp, J.A., and Post, J.A. (2002). C11-BODIPY(581/591), an oxidation-sensitive fluorescent lipid peroxidation probe: (micro) spectroscopic characterization and validation of methodology. Free Radical Bio. Med. *33*, 473-490.

S15. Johnson, L., Mulcahy, H., Kanevets, U., Shi, Y., and Lewenza, S. (2012). Surface-localized spermidine protects the *Pseudomonas aeruginosa* outer membrane from antibiotic treatment and oxidative stress. J. Bacteriol. *194*, 813-826.

S16. Pap, E.H., Drummen, G.P., Winter, V.J., Kooij, T.W., Rijken, P., Wirtz, K.W., Op den Kamp, J.A., Hage, W.J., and Post, J.A. (1999). Ratio-fluorescence microscopy of lipid oxidation in living cells using C11-BODIPY(581/591). FEBS Lett. *453*, 278-282.
