# Supplementary figures and images for: Cell Growth of Wall-Free L-Form Bacteria Is Limited by Oxidative Damage
Source: Curr Biol. 2015 Jun 15;25(12):1613–8. doi: 10.1016/j.cub.2015.04.031 (PMC4510147; doi:10.1016/j.cub.2015.04.031)

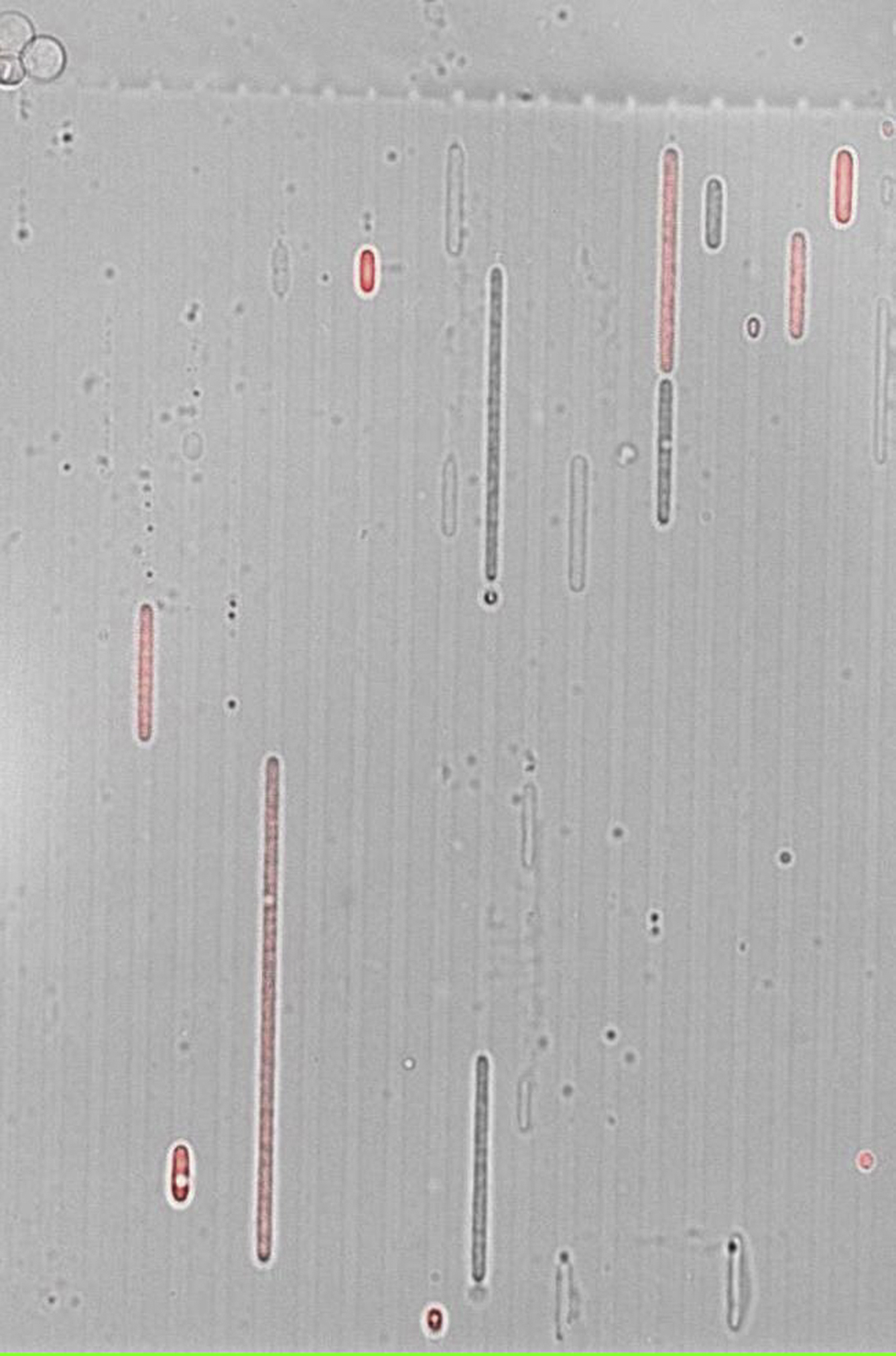

Supplement: Movie S1. Effects of ispA Mutation in Protoplast Growth, Related to Figure 1B — Time-lapse series with a microfluidic system showing the growth of protoplasts of strains BS115 (Pxyl-murE) and 4738 (Pxyl-murE ispA∗ aprE::PrpsD-mcherry) in NB and MSM (no xylose), from which the panels in Figure 1B were obtained. Phase contrast and the corresponding mCherry images were acquired automatically every 5 min. [file mmc3.jpg]
