# Supplementary material for: Cell Growth of Wall-Free L-Form Bacteria Is Limited by Oxidative Damage
Source: Curr Biol. 2015 Jun 15;25(12):1613–8. doi: 10.1016/j.cub.2015.04.031 (PMC4510147; doi:10.1016/j.cub.2015.04.031)
Supplement: Document S2. Article plus Supplemental Information [file mmc4.pdf]

Current Biology

Report

Cell Growth of Wall-Free L-Form Bacteria Is Limited by Oxidative Damage

Highlights

- The cellular levels of ROS are increased when cell wall synthesis is blocked
- Oxidative damage is a serious impediment to growth of wall-deficient L-forms
- Reduction of ROS levels promotes L-form growth
- L-forms provide new insights into the mode of action of cell wall antibiotics

Authors

Yoshikazu Kawai, Romain Mercier, Ling Juan Wu, Patricia Domínguez-Cuevas, Taku Oshima, Jeff Errington

Correspondence

y.kawai@ncl.ac.uk (Y.K.),
jeff.errington@ncl.ac.uk (J.E.)

In Brief

Although many bacteria are capable of switching into a cell-wall-deficient L-form state, the molecular basis of the transition is still poorly understood. Kawai et al. show that the cellular levels of ROS are abnormally increased when cell wall synthesis is blocked and that counteraction of ROS production leads to improved L-form growth.

Kawai et al., 2015, Current Biology *25*, 1613–1618
June 15, 2015 ©2015 The Authors
http://dx.doi.org/10.1016/j.cub.2015.04.031

Current Biology
Report

Cell Growth of Wall-Free L-Form Bacteria Is Limited by Oxidative Damage

Yoshikazu Kawai,[1,4,*] Romain Mercier,[1,4] Ling Juan Wu,[1] Patricia Domínguez-Cuevas,[2] Taku Oshima,[3] and Jeff Errington[1,*]
[1]Centre for Bacterial Cell Biology, Institute for Cell and Molecular Biosciences, Medical School, Newcastle University, Richardson Road, Newcastle upon Tyne NE2 4AX, UK
[2]Department of Biology, University of Copenhagen, Ole Maaløes Vej 5, 2200 Copenhagen N, Denmark
[3]Genomics of Bacterial Cell Functions, Graduate School of Biological Sciences, Nara Institute of Science and Technology, 8916-5, Takayama, Ikoma, Nara 630-0192, Japan
[4]Co-first author
*Correspondence: y.kawai@ncl.ac.uk (Y.K.), jeff.errington@ncl.ac.uk (J.E.)
http://dx.doi.org/10.1016/j.cub.2015.04.031
This is an open access article under the CC BY license (http://creativecommons.org/licenses/by/4.0/).

SUMMARY

The peptidoglycan (PG) cell wall is a defining feature of the bacterial lineage and an important target for antibiotics, such as β-lactams and glycopeptides. Nevertheless, many bacteria are capable of switching into a cell-wall-deficient state, called the "L-form" [1–3]. These variants have been classically identified as antibiotic-resistant forms in association with a wide range of infectious diseases [4]. L-forms become completely independent of the normally essential FtsZ cell division machinery [3, 5]. Instead, L-form proliferation is driven by a simple biophysical process based on an increased ratio of surface area to cell volume synthesis [6, 7]. We recently showed that only two genetic changes are needed for the L-form transition in *Bacillus subtilis* [7]. Class 1 mutations work to generate excess membrane synthesis [7]. Until now, the function of the class 2 mutations was unclear. We now show that these mutations work by counteracting an increase in the cellular levels of reactive oxygen species (ROS) originating from the electron transport pathway, which occurs in wall-deficient cells. Consistent with this, addition of a ROS scavenger or anaerobic culture conditions also worked to promote L-form growth without the class 2 mutations in both Gram-positive *B. subtilis* and Gram-negative *Escherichia coli*. Our results suggest that physiological compensation for the metabolic imbalance that occurs when cell wall synthesis is blocked is crucial for L-form proliferation in a wide range of bacteria and also provide new insights into the mode of action of antibiotics that target the bacterial cell wall.

RESULTS

The *ispA* Mutation Suppresses Cell Lysis during Protoplast Growth

We previously showed that protoplasts of *B. subtilis*, derived by stripping the cell wall with lysozyme in the presence of an osmoprotective agent such as sucrose (Figure 1A, a), fail to proliferate in this state [7]. However, mutants able to proliferate, called "L-forms," can be selected, and this requires a combination of two kinds of mutations [5, 7]. Class 1 mutations are of several different types, but all work by generating excess amounts of cell membrane, which drives spontaneous shape changes and ultimately proliferation (Figure 1A, b). The original class 2 mutation, which lay in a gene called *ispA* [5], seemed to work by stabilizing the proliferating L-forms and preventing them from lysing [7] (Figure 1A, c and d). For reasons that remain unclear, mutations that block cell wall precursor synthesis have a class 1 phenotype (i.e., generate excess cell membrane), and this is convenient because these mutations simultaneously prevent the cell wall from being restored.

To improve our understanding of the effects of the *ispA* mutation on cell lysis, we took advantage of recently developed microfluidic methods [8]. In channels of the microfluidic system, protoplasts are constrained into a near-typical rod-shaped morphology, with approximately similar width to that of wild-type walled cells. Figure 1B (and Movie S1) shows a mixture of *B. subtilis* protoplasts containing a repressible P_{xyl}-*murE* construct (acts as a class 1 mutation) with (*ispA*$^{-}$; red cells expressing mCherry) or without (*ispA*$^{+}$; unlabelled cells) an *ispA* mutation trapped in the channels. The *ispA*$^{-}$ mutant protoplasts (red cells) mainly grew well over many hours in L-form medium in the absence of xylose. In contrast, *ispA*$^{+}$ protoplasts (unlabelled cells) frequently lysed after only a limited amount of growth (94% of *ispA*$^{+}$ [n = 36] and 35% of *ispA*$^{-}$ protoplasts [n = 23] resulted in cell lysis in similar experiments). Thus, protoplasts in which peptidoglycan (PG) synthesis is inhibited tend to lyse even in the absence of L-form-like shape changes and cell division, and this cell lysis is suppressed by an *ispA* mutation.

Reduction of Electron Transport Chain Activity Promotes L-Form Growth

IspA catalyzes the formation of farnesyl pyrophosphate (FPP) in the polyprenoid synthetic pathway [9] (Figure 2A). This pathway leads to the formation of two lipid molecules: heptaprenyl diphosphate (HPP), required for synthesis of menaquinone (MQ), which is involved in the electron transport chain (ETC) system, and undecaprenyl pyrophosphate (UPP), required for synthesis of the precursors for peptidoglycan (lipid II) and wall teichoic acid. If the *ispA* mutation works through one of these

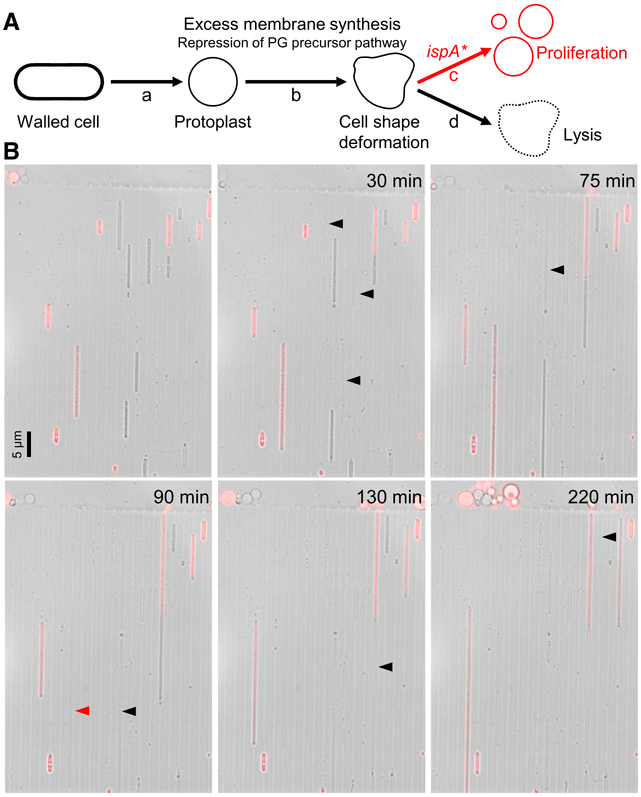

Figure 1. Effects of *ispA* Mutation and Repression of PG Precursor Pathway in Protoplast Growth

(A) Schematic representation of the requirements for cell proliferation in cell-wall-free *B. subtilis*. See text for details.

(B) *B. subtilis* strains, BS115 (P_{xyl}-*murE*) and 4738 (P_{xyl}-*murE ispA* aprE*::P_{rpsD}-*mcherry*), were grown in the walled state (with xylose), then converted to protoplasts, and incubated in nutrient broth (NB) and magnesium-sucrose-maleic acid (MSM) (no xylose) with PenG. After 5 hr, the cultures were mixed, and the cells were observed by time-lapse microscopy via a microfluidic system. Representative images are shown of overlays of the phase contrast (PC) and corresponding mCherry signals. Elapsed time (min) is shown in each panel. Lysed cells are labeled with arrowheads. The scale bar represents 5 µm. See also Movie S1.

pathways, repression of either *hepS* (encoding HPP synthase) or *uppS* (encoding UPP synthase) should also promote L-form growth. Figure 2B shows a comparison of the effects of repression (isopropyl β-D-1-thiogalactopyranoside (IPTG)-dependent P_{spac} constructs) of *hepS*, *uppS*, or *ispA* on growth of walled cells or L-forms. When grown as walled cells, all three strains grew similarly to the wild-type in the presence of IPTG (left plate). In the absence of IPTG (center plate) neither the *hepS* nor the *uppS* repression strain grew, consistent with the essential roles for UppS and HepS in walled cells [11]. Repression of *ispA* did not suppress growth, presumably because it has a paralog, *hepT*, which encodes a component of HPP synthase [11]. We then induced L-form growth by inhibiting the PG precursor pathway with D-cycloserine (DCS), which inhibits the D-alanine-D-alanine ligase, Ddl (see [3]) (the FtsZ-targeting antibiotic 8j was added to kill walled cells; [7]). As expected, repression of *ispA* enabled growth on the L-form selective plates (right-hand panel of Figure 2B). Repression of *hepS* also supported growth on L-form plates, but *uppS* did not. Phase contrast microscopy confirmed the presence of heterogenous spheroidal cells on the *hepS* repression plate, similar to the L-forms induced by *ispA* repression (Figure 2C). These results suggest that the effect of the *ispA* mutation on L-form growth operates through the HPP/MQ pathway rather than the lipid II pathway.

In previous experiments selecting de novo L-form variants, we repeatedly found that the most robust colonies tended to have mutations in the *ispA* gene [5, 7]. To find out whether mutations in genes other than *ispA* would support L-form growth, we performed a screen in cells containing a second copy of *ispA*. A transposon mutant library was made in cells containing P_{xyl}-*murE* (class 1 mutation) grown in the walled state (presence of xylose). L-forms were selected from the mutant library on plates containing sucrose as an osmoprotectant (but no xylose) and the cell division inhibitor, 8j. Six independent transposon mutants were selected, checked by backcrossing, and the sites of transposon insertion were determined by sequencing. Two mutations lay in each of the *ndh* and *qoxB* genes, and single insertions were found in *ctaB* and *mhqR* (Figures 2D and 2E). As shown in Figure 2A, three of these genes encode products involved in the ETC system: *ndh* encodes a major NADH dehydrogenease [12]; *qoxB* encodes cytochrome aa_3 quinol oxidase subunit I [13]; *ctaB* encodes heme O synthase [14]; and *mhqR* encodes a transcriptional repressor for genes induced by the thiol-specific oxidative and/or electrophile stress response [15]. Figures 2D and 2E show the enabling effects of these mutations on L-form growth. Taken collectively, these results suggest that L-form growth, under the conditions we normally use, requires a reduction of ETC activity, and the identification of an *mhqR* mutation further suggests that reactive oxygen species (ROS) originating from the ETC pathway prevent L-form growth. For reasons that are not yet clear, L-forms generated by either *ispA* or *mhqR* mutations were able to grow in liquid medium, whereas the others would only grow on solid agar plates (data not shown).

Abnormally Increased Cellular ROS Levels in Protoplasts Are Reduced by Switching into the L-Form State

In general, all aerobic organisms use oxygen as the terminal electron acceptor for efficient energy production. However, ROS is also generated as a by-product through the metabolism of molecular oxygen, and this causes damage to nucleotides, proteins, and lipids [16, 17]. The cell therefore has various genetic systems to respond to oxidative stress. To investigate whether the oxidative stress response was induced in protoplasts, we compared the gene expression patterns of protoplasts (P_{xyl}-*murE*, with and without xylose) to those of walled cells (P_{xyl}-*murE*, with xylose) and L-forms (P_{xyl}-*murE ispA**, without xylose) using microarrays. The results showed that the transcription of 103 genes was specifically induced in protoplasts (Tables S1 and S2). Many of those genes (43 genes) have roles against various stresses, including resistance to oxidative and electrophile stress (13 genes), cell envelope stress (12 genes), and heat shock (6 genes). As shown in Figure 3A (see also Figure S1A), strong induction of genes belonging to the PerR regulon, which is induced by the

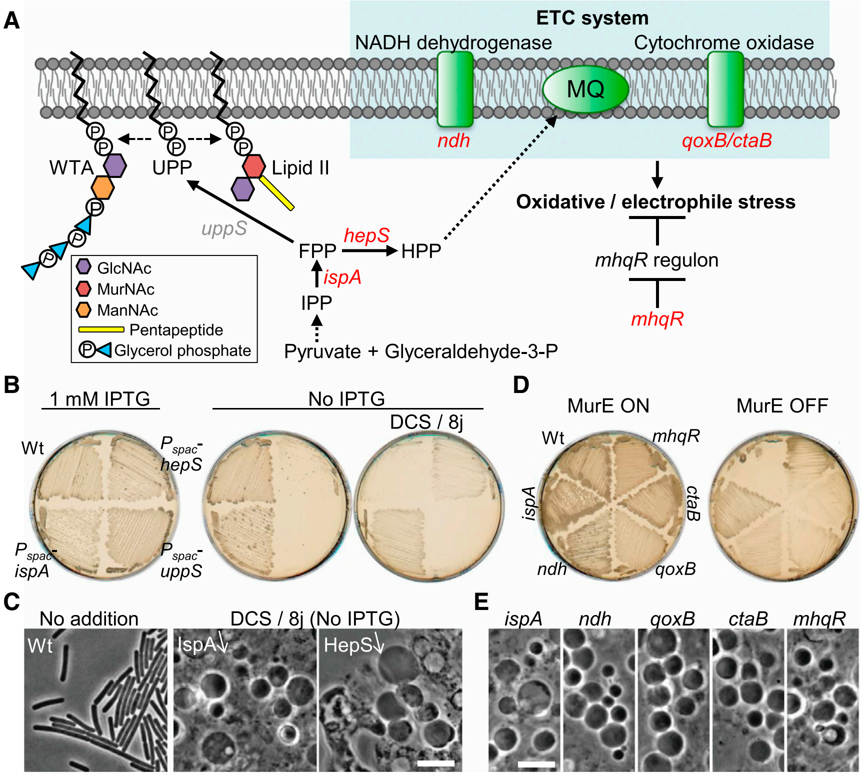

Figure 2. Effects of ETC Activity on L-Form Growth

(A) Schematic representation of the UPP and HPP synthetic pathways and ETC system in *B. subtilis*. Repression of various genes (indicated in red) supports L-form growth when combined with inhibition of the PG precursor pathway. See text for details.

(B) Effects of repression of *ispA*, *uppS*, and *hepS* on cell growth in the absence of a cell wall. *B. subtilis* strains, wild-type (Wt; 168CA), YK1424 (P_{spac}-*ispA*), YK1889 (Δ*uppS* pLOSS-P_{spac}-*uppS* P_{xyl}-*cdsA*), and YK1450 (P_{spac}-*hepS*) were grown on nutrient agar (NA) and MSM plates containing 1% xylose with or without 1 mM IPTG and 400 μg/ml DCS (with 1 μg/ml 8j to prevent the rare reversion to walled cells) at 30°C. Note that the *uppS* lies immediately upstream of *cdsA*, which is essential for membrane phospholipid synthesis. To avoid the polar effect on the *cdsA* expression, we inserted a xylose-inducible promoter (P_{xyl}) in front of the *cdsA* gene, as described previously [10].

(C) PC micrographs of wild-type walled cells and L-forms (P_{spac}-*ispA* and P_{spac}-*hepS*) taken from the cultures shown in (B). The scale bar represents 5 μm.

(D) Effects of transposon mutations on cell growth in the absence of a cell wall. *B. subtilis* strains, BS115 (Wt; P_{xyl}-*murE*), LR2 (*ispA*; P_{xyl}-*murE ispA**), YK1816 (*ndh*; P_{xyl}-*murE ndh*::*tn*), YK1817 (*qoxB*; P_{xyl}-*murE qoxB*::*tn*), YK1818 (*ctaB*; P_{xyl}-*murE ctaB*::*tn*), and YK1522 (*mhqR*; P_{xyl}-*murE mhqR*::*tn*) were grown on NA and MSM plates with (MurE ON) and without (MurE OFF) 2% xylose at 30°C for 2 days (MurE ON) and 3 days (MurE OFF).

(E) PC micrographs of L-forms taken from the cultures shown in (D). The scale bar represents 5 μm.

peroxide-induced oxidative stress [18], was detected in protoplasts. In comparison, many essential genes or functions, such as DNA replication and protein synthesis, for the growth of normal walled state were downregulated in protoplasts (Table S1). The stringent response (Figure S1B), which is induced by amino acid starvation or other stresses [19], may be largely responsible for these downregulation effects. To confirm induction of the oxidative stress response in protoplasts, we examined expression of the *katA* promoter using a P_{katA}-*gfp* fusion [20]. The *katA* gene encodes a vegetative catalase and is part of the PerR regulon. Figure 3B shows a mixture of exponentially growing wild-type walled cells and an overnight culture of protoplasts with PG precursor synthesis repressed (P_{xyl}-*murE*, no xylose) in L-form medium. An obvious GFP signal was detected in most protoplasts, whereas little or no detectable GFP fluorescence was seen in the walled cells (Figure 3B, GFP). We then examined the P_{katA} activity in a mixture of the protoplasts (unlabelled) and L-forms (P_{xyl}-*murE ispA* aprE*::*mCherry*, red cells expressing mCherry). Whereas strong GFP signals were present in many protoplasts, the GFP signals in L-forms were much weaker (Figure 3C). The result is consistent with oxidative stress being significantly induced in protoplasts, but not in L-forms.

To assay more directly for ROS production in protoplasts, we took advantage of a fluorescent fatty acid analog, C_{11}-BODIPY$^{581/591}$, which has been used as an indicator of oxidative damage to lipids, i.e., lipid peroxidation [21, 22]. The probe is incorporated into membranes, and the fluorescent properties in the red range of the visible spectrum (emission maximum 595 nm) in fluorescence microscopy shift to the green range (520 nm) upon free radical-induced oxidation. In a control experiment with wild-type *B. subtilis* walled cells exponentially growing in sucrose-based L-form medium, the typical rod-shaped cells exhibited a highly regular and smooth red fluorescence at the cell surface, but no clear green fluorescence was detectable (Figure 3D, i). After treatment with H_2O_2, green fluorescence was evident within most cells, and the staining was patchy and irregular (Figure 3D, ii). In contrast to the walled cells, green fluorescence was readily detectable in protoplasts (P_{xyl}-*murE*, no xylose) in the absence of exogenous oxidant (Figure 3E), suggesting lipid peroxidation by endogenous oxygen radicals. Importantly, the fluorescent shift was largely suppressed in L-forms carrying an *ispA* mutation (P_{xyl}-*murE ispA**, no xylose) (Figures 3F and 3G). Thus, the cellular ROS levels are indeed increased in protoplasts, but they are suppressed by reduction of the ETC activity via *ispA* mutation. Consistent with the idea that the increased cellular ROS levels in protoplasts originate from the ETC pathway, strong induction of various genes involved in the tricarboxylic acid (TCA) cycle was detected in protoplasts by microarray experiments (Figures S1C and S1D).

Oxidative Stress Response Genes Are Required to Support L-Form Growth

As described above, microarray experiments showed specific induction of genes in the PerR regulon and other genes for resistance against oxidative stress in protoplasts (Figure S1A).

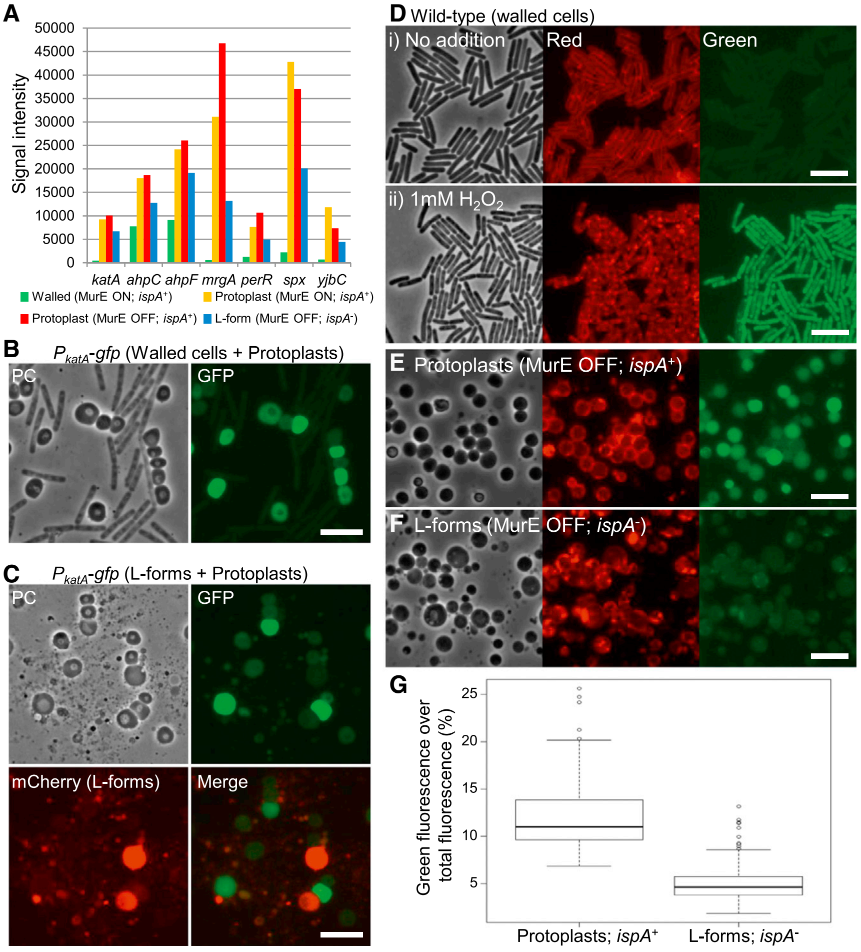

Figure 3. Increased ROS Production in Protoplasts and Its Suppression by an *ispA* Mutation

(A) Expression patterns of several PerR regulated genes cultured in the walled (green; strain BS115; P_{xyl}-*murE*, 2% xylose), protoplast (yellow and red; strain BS115; P_{xyl}-*murE*, 2% or no xylose), or L-form (blue; strain LR2; P_{xyl}-*murE ispA**, no xylose) states. See also Figure S1A and Table S1.

(B) PC micrograph of a mixture of exponentially growing *B. subtilis* walled cells (YK2003; P_{xyl}-*murE amyE*::P_{katA}-*gfp*, 2% xylose) and an overnight culture of protoplasts (YK2003; no xylose) in NB and MSM (PC). The right panel shows the corresponding GFP image. The scale bar represents 5 μm.

(C) Effect of *ispA* mutation on P_{katA} activity in protoplasts. PC micrograph of a mixture of overnight cultures of protoplasts (YK2003; P_{xyl}-*murE amyE*::P_{katA}-*gfp*, no xylose) and L-forms (YK2005; P_{xyl}-*murE ispA* amyE*::P_{katA}-*gfp aprE*::P_{rpsD}-*mcherry*, no xylose) in NB and MSM (PC). The corresponding epifluorescence images of GFP and mCherry and the merge of GFP and mCherry are shown, respectively. The scale bar represents 5 μm.

(D–F) The fluorescent probe C_{11}-BODIPY$^{581/591}$ was used as an indicator of lipid peroxidation. The probe undergoes a shift from red to green fluorescence emission upon peroxidation (see Supplemental Experimental Procedures). The scale bar represents 5 μm.

(D) PC and corresponding epifluorescence micrographs (red and green fluorescence channels) of *B. subtilis* wild-type strain 168CA, grown in NB and MSM with (ii) and without (i) 1 mM H_2O_2.

(E and F) PC and corresponding epifluorescence micrographs (red and green fluorescence channels) of overnight cultures of protoplasts (BS115; P_{xyl}-*murE*, E) or L-forms (LR2; P_{xyl}-*murE ispA**, F) in NB and MSM without xylose.

(G) The relative signal intensity of green fluorescence over total fluorescence (green + red fluorescence) of protoplasts (left; *ispA*$^+$ [n = 201]) and L-forms (right; *ispA*$^-$ [n = 199]). The signal intensity was obtained from similar images to (E) and (F) by ImageJ. Boxplots represent median (horizontal black lines), the upper and lower quartile values (boxes), and the most extreme data points within 1.5 times interquartile ranges (whiskers). The tendency of the weakness on the green fluorescent intensity in the L-forms is statistically significant (Student's t test, $p < 0.01$). Student's t test and the preparation of a boxplot were performed by the statistics software package, R.

However, in many cases, the expression levels in L-forms were also significantly higher than those of walled cells. We wondered whether the stress response genes might be needed to protect cells against oxidative stress during L-form growth. To test this, we inserted an IPTG-dependent promoter in front of four genes or operons encoding antioxidant systems, *katA* (main vegetative catalase) [23], *sodA* (superoxide dismutase) [24], *bshB1/bshA* (bacillithiol synthesis) [25], and *zwf* (glucose 6-phosphate dehydrogenase; for NADPH generation) [26], in strain LR2 (P_{xyl}-*murE ispA**). Note that the thioredoxin system is essential for viability in walled cells and that *B. subtilis* lacks a glutathione (GSH) system. In the walled state, none of the mutations had a significant effect on cell growth (Figure 4A, MurE ON/No IPTG). However, in L-form state (MurE OFF/No IPTG), repression of the *sodA*, *bshB1* operon, or *zwf* genes severely inhibited growth. Repression of *katA* did not abolish L-form growth, but this was not completely surprising as *B. subtilis* has several paralogous genes and an alkyl hydroperoxide reductase [27]. Induction of the antioxidant systems by addition of IPTG restored growth in each case (Figure 4A, MurE OFF/1 mM IPTG). Thus, several antioxidant systems seem to be crucial for L-form proliferation in *B. subtilis*.

Reduction of ROS Promotes L-Form Growth in *E. coli*

We have previously reported that L-forms of the Gram-negative bacterium *E. coli* do not require an *ispA*-like mutation for proliferation on L-form plates (containing sucrose as osmoprotectant and fosfomycin, an inhibitor of the PG precursor pathway) [3]. Nevertheless, since the growth of *E. coli* L-forms is apparently much slower than that of *B. subtilis*, we wondered whether this was again due to oxidative damage. If so, then treatment of cells with a ROS scavenger such as reduced GSH might improve the growth of *E. coli* L-forms. *E. coli* walled cells were streaked on L-form selective plates with and without GSH (Figure 4B, i). In the absence of GSH, discrete L-form colonies were barely visible after 3 days of incubation, though a lawn of visible colonies

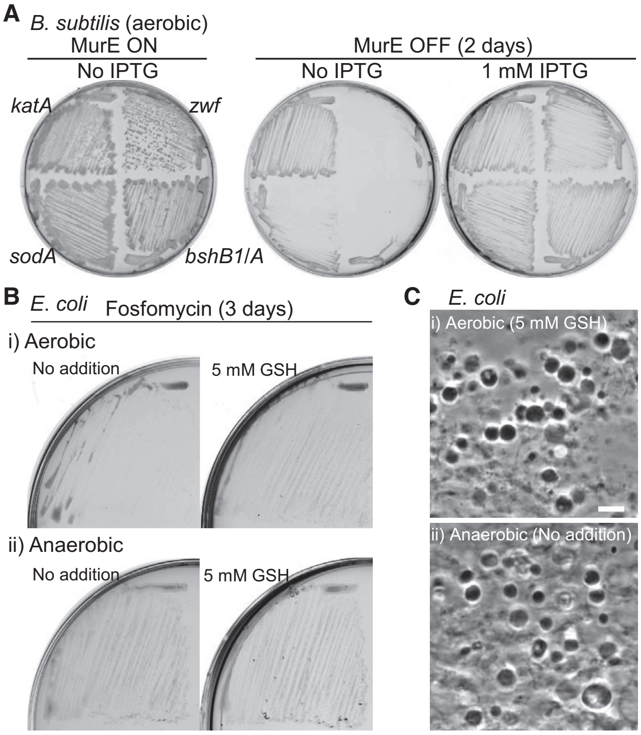

Figure 4. Effects of ROS on L-Form Growth

(A) Effect of repression of antioxidant systems on *B. subtilis* L-form growth in aerobic conditions. *B. subtilis* strains with the following mutations were cultured on NA and MSM plates with (MurE ON) or without (MurE OFF) 2% xylose at 30°C for 2 days in the presence or absence of 1 mM IPTG: P_{xyl}-*murE ispA** with P_{spac}-*katA* (YK2027), P_{spac}-*sodA* (YK2028), P_{spac}-*bshB1/A* (YK1604), and P_{spac}-*zwf* (YK1584).

(B) *E. coli* L-form growth on L-form plates (NB and MSM 1% agar with 400 μg/ml fosfomycin) at 30°C for 3 days. Growth of the *E. coli* strain RM345 (*ΔmurA* containing the unstable plasmid pOU82-*murA* [3] on L-form plates with or without 5 mM reduced GSH in aerobic (i) or anaerobic (ii) conditions. See also Figure S2.

(C) PC micrograph of *E. coli* L-forms taken from the cultures shown in (B). The scale bar represents 5 μm.

emerged after about 5 days (Figures S2A and S2B). In contrast, in the presence of GSH, significant L-form growth was seen within 3 days (Figure 4B, i; Figure 4C, i), supporting the idea that a reduction of cellular ROS levels promotes *E. coli* L-form growth. If the cellular ROS levels are increased through the metabolism of molecular oxygen in the ETC pathway in *E. coli* cell-wall-deficent cells, then anaerobic culture should also promote the growth of L-forms. Strikingly, under anaerobic conditions, significant *E. coli* L-form growth was seen within 3 days even in the absence of GSH (Figure 4B, ii; Figure 4C, ii). We also examined the effects of an *ispA* mutation on *B. subtilis* L-form growth under anaerobic condition and found that *ispA* muation was no longer required for L-form growth when oxygen was depleted (Figures S2C and S2D).

DISCUSSION

We have proposed that L-form proliferation may provide insights into an ancient mechanism used in primordial cells before the invention of the cell wall [3, 7, 28]. In this report, we have found that the cellular ROS levels are abnormally increased in cell-wall-deficient cells and that a reduction of cellular ROS levels by repression of the ETC activity, addition of a ROS scavenger or anaerobic culture, promotes the growth of wall-free L-forms in both Gram-positive and Gram-negative bacteria. Therefore, oxidative damage could be an important impediment to L-form growth in a wide range of bacteria.

Why should the L-form transition or growth in the absence of cell wall result in increased oxidative damage? Cell wall synthesis is probably a major drain on cellular resources under normal conditions, so a block in cell wall synthesis probably leads to major changes in cell metabolism. Uridine 5′-diphospho-*N*-acetyl-D-glucosamine (UDP-GlcNAc), which is an essential cell wall precursor for both lipid II and WTA synthesis (Figure 2A), is generated from fructose-6-phospate via central carbon metabolism, through the action of the *glmS*, *glmM*, and *gcaD* gene products. The *glmS* riboswitch is a ribozyme that self-cleaves upon binding glucosamine-6-phosphate, the product of the enzyme encoded by *glmS* [29]. Inhibition of PG synthesis would result in increased cellular levels of glucosamine-6-phosphate, and the subsequent repression of *glmS* could increase glycolytic flux due to a reduction in utilization of fructose-6-phospate for UDP-GlcNAc synthesis. This could, in turn, result in an increase of cellular pyruvate levels and stimulate flux into the TCA cycle. Consistent with this, our results suggest that the stimulation of ROS production in wall-deficient cells is most likely due to an increase in TCA cycle flux (see Figures S1C and S1D), leading to increased synthesis of NADH and $FADH_2$, which are the major substrates for the ETC pathway. The subsequent stimulation of ETC flux results in an increase of ROS generation as a by-product of the metabolism of molecular oxygen. Importantly, Kohanski et al. [30] have proposed a model that bactericidal antibiotics, including cell wall antibiotics, work at least in part by stimulating ROS production through a burst of NADH comsumption by the ETC pathway, although this is currently controversial [31–33]. Nevertheless, our results suggest that physiological compensation for the metabolic imbalance that occurs when the normal large flux to cell wall synthesis is blocked is crucial for the proliferation of cell-wall-free L-form bacteria. Apart from the importance for understanding early forms of cellular life, the ability to grow without a cell wall in L-forms also provides new insights into the mode of action of antibiotics that target the bacterial cell wall.

EXPERIMENTAL PROCEDURES

Experimental Procedures are described in the Supplemental Information.

ACCESSION NUMBERS

The accession number for the microarray data reported in this paper is ArrayExpress: E-MTAB-3380.

SUPPLEMENTAL INFORMATION

Supplemental Information includes Supplemental Experimental Procedures, two figures, two tables, and one movie and can be found with this article online at http://dx.doi.org/10.1016/j.cub.2015.04.031.

AUTHOR CONTRIBUTIONS

Y.K., R.M., and J.E. designed the experiments. Y.K., R.M., L.J.W., P.D.-C., and T.O. performed the experiments. Y.K., R.M., and T.O. analyzed the data. Y.K. and J.E. wrote the manuscript.

ACKNOWLEDGMENTS

We are very grateful to Anil Wipat and Sunny Park for establishing and providing access to the microfluidic systems. We thank Heath Murray for comments on the manuscript and Simon Syvertsson for a *B. subtilis* strain, Bss307. This work was funded by a European Research Council grant 250363 to J.E.

Received: March 3, 2015
Revised: April 9, 2015
Accepted: April 14, 2015
Published: June 4, 2015

REFERENCES

1. Klieneberger, E. (1935). The natural occurrence of pleuropneumonia-like organisms in apparent symbiosis with *Streptobacillus moniliformis* and other bacteria. J. Pathol. Bacteriol. *40*, 93–105.
2. Allan, E.J., Hoischen, C., and Gumpert, J. (2009). Bacterial L-forms. Adv. Appl. Microbiol. *68*, 1–39.
3. Mercier, R., Kawai, Y., and Errington, J. (2014). General principles for the formation and proliferation of a wall-free (L-form) state in bacteria. eLife *3*, e04629.
4. Domingue, G.J., Sr., and Woody, H.B. (1997). Bacterial persistence and expression of disease. Clin. Microbiol. Rev. *10*, 320–344.
5. Leaver, M., Domínguez-Cuevas, P., Coxhead, J.M., Daniel, R.A., and Errington, J. (2009). Life without a wall or division machine in *Bacillus subtilis*. Nature *457*, 849–853.
6. Mercier, R., Domínguez-Cuevas, P., and Errington, J. (2012). Crucial role for membrane fluidity in proliferation of primitive cells. Cell Rep. *1*, 417–423.
7. Mercier, R., Kawai, Y., and Errington, J. (2013). Excess membrane synthesis drives a primitive mode of cell proliferation. Cell *152*, 997–1007.
8. Moffitt, J.R., Lee, J.B., and Cluzel, P. (2012). The single-cell chemostat: an agarose-based, microfluidic device for high-throughput, single-cell studies of bacteria and bacterial communities. Lab Chip *12*, 1487–1494.
9. Julsing, M.K., Rijpkema, M., Woerdenbag, H.J., Quax, W.J., and Kayser, O. (2007). Functional analysis of genes involved in the biosynthesis of isoprene in *Bacillus subtilis*. Appl. Microbiol. Biotechnol. *75*, 1377–1384.
10. Kawai, Y., Mercier, R., and Errington, J. (2014). Bacterial cell morphogenesis does not require a preexisting template structure. Curr. Biol. *24*, 863–867.
11. Kobayashi, K., Ehrlich, S.D., Albertini, A., Amati, G., Andersen, K.K., Arnaud, M., Asai, K., Ashikaga, S., Aymerich, S., Bessieres, P., et al. (2003). Essential *Bacillus subtilis* genes. Proc. Natl. Acad. Sci. USA *100*, 4678–4683.
12. Gyan, S., Shiohira, Y., Sato, I., Takeuchi, M., and Sato, T. (2006). Regulatory loop between redox sensing of the NADH/NAD(+) ratio by Rex (YdiH) and oxidation of NADH by NADH dehydrogenase Ndh in *Bacillus subtilis*. J. Bacteriol. *188*, 7062–7071.
13. Santana, M., Kunst, F., Hullo, M.F., Rapoport, G., Danchin, A., and Glaser, P. (1992). Molecular cloning, sequencing, and physiological characterization of the qox operon from *Bacillus subtilis* encoding the aa3-600 quinol oxidase. J. Biol. Chem. *267*, 10225–10231.
14. Mogi, T. (2009). Over-expression and characterization of *Bacillus subtilis* heme O synthase. J. Biochem. *145*, 669–675.
15. Töwe, S., Leelakriangsak, M., Kobayashi, K., Van Duy, N., Hecker, M., Zuber, P., and Antelmann, H. (2007). The MarR-type repressor MhqR (YkvE) regulates multiple dioxygenases/glyoxalases and an azoreductase which confer resistance to 2-methylhydroquinone and catechol in *Bacillus subtilis*. Mol. Microbiol. *66*, 40–54.
16. Møller, I.M. (2001). PLANT MITOCHONDRIA AND OXIDATIVE STRESS: Electron Transport, NADPH Turnover, and Metabolism of Reactive Oxygen Species. Annu. Rev. Plant Physiol. Plant Mol. Biol. *52*, 561–591.
17. Imlay, J.A. (2013). The molecular mechanisms and physiological consequences of oxidative stress: lessons from a model bacterium. Nat. Rev. Microbiol. *11*, 443–454.
18. Helmann, J.D. (2014). Specificity of metal sensing: iron and manganese homeostasis in *Bacillus subtilis*. J. Biol. Chem. *289*, 28112–28120.
19. Boutte, C.C., and Crosson, S. (2013). Bacterial lifestyle shapes stringent response activation. Trends Microbiol. *21*, 174–180.
20. Hoover, S.E., Xu, W., Xiao, W., and Burkholder, W.F. (2010). Changes in DnaA-dependent gene expression contribute to the transcriptional and developmental response of *Bacillus subtilis* to manganese limitation in Luria-Bertani medium. J. Bacteriol. *192*, 3915–3924.
21. Drummen, G.P., van Liebergen, L.C., Op den Kamp, J.A., and Post, J.A. (2002). C11-BODIPY(581/591), an oxidation-sensitive fluorescent lipid peroxidation probe: (micro)spectroscopic characterization and validation of methodology. Free Radic. Biol. Med. *33*, 473–490.
22. Johnson, L., Mulcahy, H., Kanevets, U., Shi, Y., and Lewenza, S. (2012). Surface-localized spermidine protects the *Pseudomonas aeruginosa* outer membrane from antibiotic treatment and oxidative stress. J. Bacteriol. *194*, 813–826.
23. Chen, L., Keramati, L., and Helmann, J.D. (1995). Coordinate regulation of *Bacillus subtilis* peroxide stress genes by hydrogen peroxide and metal ions. Proc. Natl. Acad. Sci. USA *92*, 8190–8194.
24. Casillas-Martinez, L., and Setlow, P. (1997). Alkyl hydroperoxide reductase, catalase, MrgA, and superoxide dismutase are not involved in resistance of Bacillus subtilis spores to heat or oxidizing agents. J. Bacteriol. *179*, 7420–7425.
25. Gaballa, A., Newton, G.L., Antelmann, H., Parsonage, D., Upton, H., Rawat, M., Claiborne, A., Fahey, R.C., and Helmann, J.D. (2010). Biosynthesis and functions of bacillithiol, a major low-molecular-weight thiol in Bacilli. Proc. Natl. Acad. Sci. USA *107*, 6482–6486.
26. Prieto-Alamo, M.J., Jurado, J., Gallardo-Madueno, R., Monje-Casas, F., Holmgren, A., and Pueyo, C. (2000). Transcriptional regulation of glutaredoxin and thioredoxin pathways and related enzymes in response to oxidative stress. J. Biol. Chem. *275*, 13398–13405.
27. Seaver, L.C., and Imlay, J.A. (2001). Alkyl hydroperoxide reductase is the primary scavenger of endogenous hydrogen peroxide in *Escherichia coli*. J. Bacteriol. *183*, 7173–7181.
28. Errington, J. (2013). L-form bacteria, cell walls and the origins of life. Open Biol. *3*, 120143.
29. Winkler, W.C., Nahvi, A., Roth, A., Collins, J.A., and Breaker, R.R. (2004). Control of gene expression by a natural metabolite-responsive ribozyme. Nature *428*, 281–286.
30. Kohanski, M.A., Dwyer, D.J., Hayete, B., Lawrence, C.A., and Collins, J.J. (2007). A common mechanism of cellular death induced by bactericidal antibiotics. Cell *130*, 797–810.
31. Liu, Y., and Imlay, J.A. (2013). Cell death from antibiotics without the involvement of reactive oxygen species. Science *339*, 1210–1213.
32. Keren, I., Wu, Y., Inocencio, J., Mulcahy, L.R., and Lewis, K. (2013). Killing by bactericidal antibiotics does not depend on reactive oxygen species. Science *339*, 1213–1216.
33. Dwyer, D.J., Collins, J.J., and Walker, G.C. (2015). Unraveling the physiological complexities of antibiotic lethality. Annu. Rev. Pharmacol. Toxicol. *55*, 313–332.

Current Biology
Supplemental Information

Cell Growth of Wall-Free L-Form Bacteria Is Limited by Oxidative Damage

Yoshikazu Kawai, Romain Mercier, Ling Juan Wu, Patricia Domínguez-Cuevas, Taku Oshima, and Jeff Errington

Figure S1

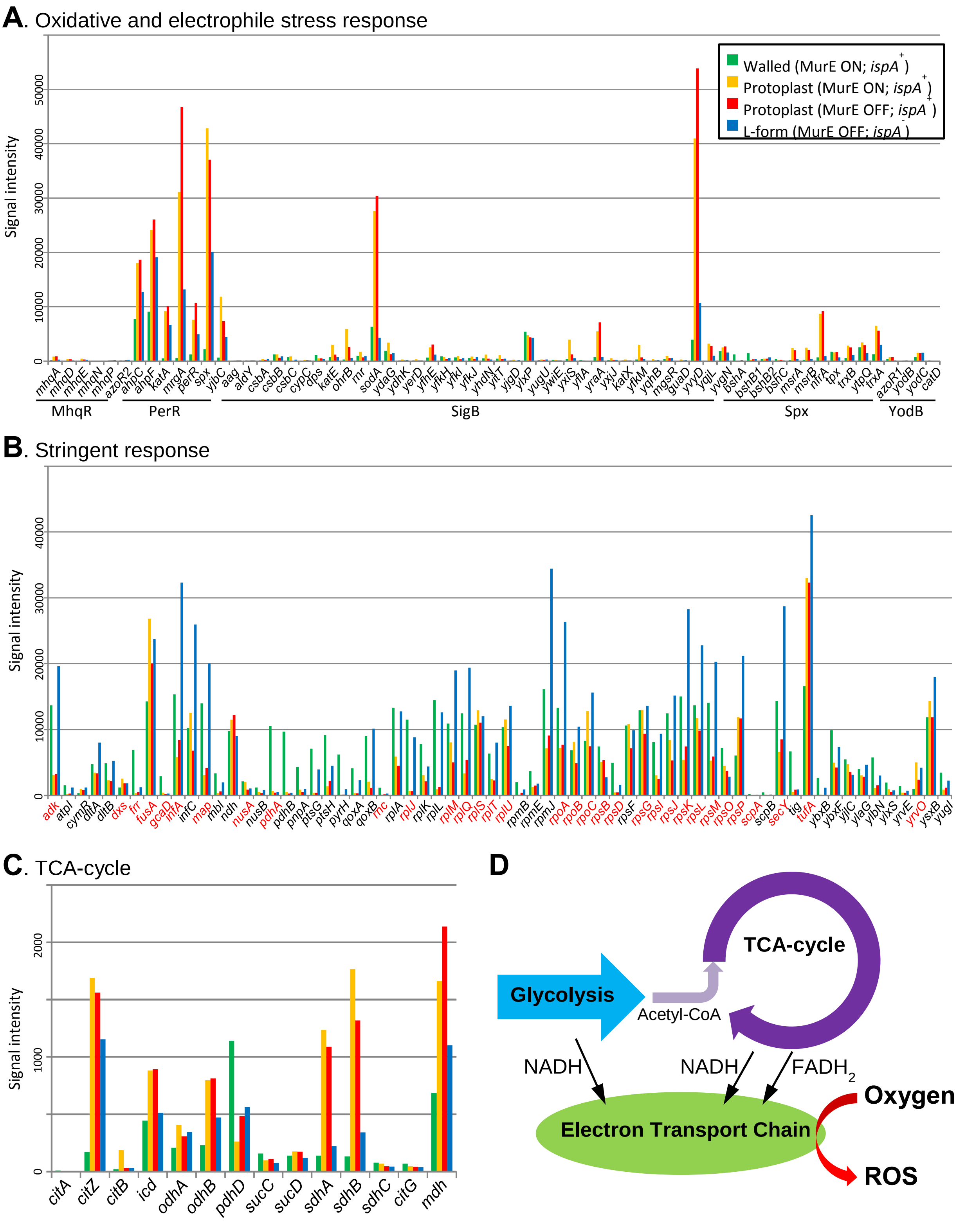
A. Oxidative and electrophile stress response
Signal intensity
Walled (MurE ON; ispA+)
Protoplast (MurE ON; ispA+)
Protoplast (MurE OFF; ispA+)
L-form (MurE OFF; ispA-)
MhqR
PerR
SigB
Spx
YodB
B. Stringent response
Signal intensity
C. TCA-cycle
Signal intensity
citA
citZ
citB
icd
odhA
odhB
pdhD
sucC
sucD
sdhA
sdhB
sdhC
citG
mdh
D
TCA-cycle
Glycolysis
Acetyl-CoA
NADH
NADH
FADH2
Electron Transport Chain
Oxygen
ROS

Figure S1, related to Figure 3. Expression patterns of various genes in protoplasts and L-forms

(A-C) Expression patterns of various genes in walled (green, strain BS115; P_{xyl}-*murE*, 2% xylose), protoplast (yellow, BS115; P_{xyl}-*murE*, 2% xylose, and red, P_{xyl}-*murE*, no xylose) or L-form (blue, LR2; P_{xyl}-*murE ispA**, no xylose) cells.

(A) Expression patterns of genes related to resistance against oxidative and electrophile stress. Their corresponding transcriptional regulators, MhqR, PerR, SigB, Spx and YodB, are shown below the genes.

(B) Expression patterns of genes affected by the stringent response. Genes essential for viability of normal walled cells are indicated in red.

(C) Expression patterns of genes in the TCA cycle.

(D) Schematic representation of the links between the TCA cycle, the ETC pathway and ROS generation. See detail in Discussion.

Figure S2

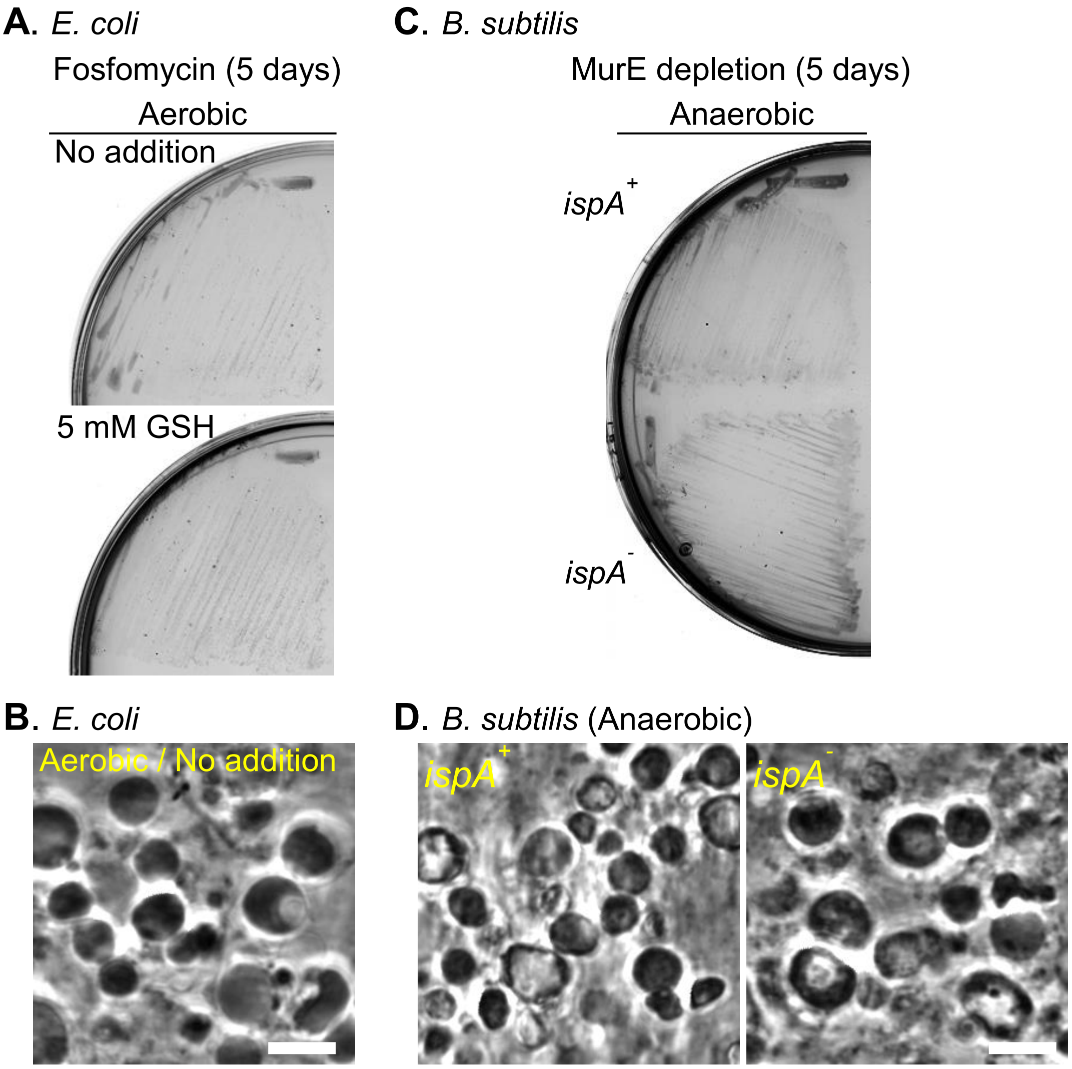
A. *E. coli*
Fosfomycin (5 days)
Aerobic
No addition
5 mM GSH
C. *B. subtilis*
MurE depletion (5 days)
Anaerobic
ispA+
ispA-
B. *E. coli*
Aerobic / No addition
D. *B. subtilis* (Anaerobic)
ispA+
ispA-

Figure S2, related to Figure 4. Effects of ROS on L-form growth

(A) Growth of the *E*. *coli* strain RM345 (*ΔmurA* and containing the unstable plasmid pOU82-*murA* [S1] on L-form plates (NB/MSM 1% agar with 400 μg/ml fosfomycin) with or without 5 mM reduced glutathione (GSH) at 30°C for 5 days under aerobic conditions.

(B) Phase contrast micrograph of *E*. *coli* L-forms taken from the culture shown in panel A (No GSH addition). Scale bar represents 5 μm.

(C) Growth of the *B*. *subtilis* strains BS115 (P_{xyl}-*murE*) and LR2 (P_{xyl}-*murE ispA**) on L-form plates (no xylose) with 10 mM Nitrate at 30°C for 5 days under anaerobic conditions. Note that *B. subtilis* was unable to grow in the absence of molecular oxygen as a terminal electron acceptor [S2].

(D) Phase contrast micrograph of *B*. *subtilis* L-forms taken from the cells shown in panel C under aerobic situation. Scale bar represents 5 μm.

Table S1. Summary of the microarray results

Function categories	P > L[a]		L > P[b]	
	P > W and L[c] (P-3hr > W)[d]	P ≒ W > L[e]	L > W and P[f]	L ≒ W > P[g]
**Carbon metabolism**	11 (7)	4	19	6
**Amino acid metabolism**	5 (5)	0	1	16
**Nucleotide metabolism**	1 (1)	2	0	4
**Lipid metabolism**	1 (0)	1	0	3
**Electron transport / ATP synthesis**	4 (1)	0	3	12
**Metal Homeostasis**	5 (4)	0	0	5
**DNA replication / segregation / repair**	1 (1)	0	0	6
**RNA and protein synthesis**	1 (1)	1	3	23
**Cell wall synthesis / cell division**	2 (1)	1	3	4
**Stress response**	43 (26)	10	16	9
**Prophages**	3 (3)	1	38	2
**Unknown function**	20 (16)	12	11	28
**Others**	6 (4)	14	1	5
**Total (number of genes)**	**103 (70)**	**46**	**95**	**123**

Total RNA was isolated from *B. subtilis* cells (OD_{600nm}=0.2~0.3) cultured in NB/MSM at 30°C (see detail in Experimental Procedures). P; overnight culture (18hr incubation) of protoplasts of strain BS115 (P_{xyl}-*murE*, no xylose), L; exponentially growing L-forms of strain LR2 (P_{xyl}-*murE ispA**, no xylose), W; exponentially growing walled cells of strain BS115 (P_{xyl}-*murE*, 2% xylose) and P-3hr; protoplasts of strain BS115 (P_{xyl}-*murE*, no xylose) incubated for 3 hr after conversion to protoplasts.

[a]Number of genes that revealed higher expression levels (more than three times) in P than L.

[b]Number of genes that revealed higher expression levels (more than three times) in L than P.

[C]Number of genes that revealed higher expression levels (more than three times) in P than W and L. (see detail in Table S2i)

[d]Number of genes that revealed higher expression levels (more than three times) in P-3hr than W.

[e]Number of genes that revealed higher expression levels (more than three times) in P than L, and no significant difference between P and W. (see detail in Table S2ii)

[f]Number of genes that revealed higher expression levels (more than three times) in L than W and P. (see detail in Table S2iii)

[g]Number of genes that revealed higher expression levels (more than three times) in L than P, and no significant difference between L and W. (see detail in Table S2iv)

Bacterial strains and plasmid used in this study

**Strain**	**Relevant genotype**	**Reference**
B. subtilis		
168CA	*trpC2*	Lab. stock
BS115	168CA Ω*spoVD*::*cat* P_{xyl}-*murE* Ω*amyE*::*xylR tet*	Lever et al., 2009 [S3]
LR2	BS115 *xseB** (Frameshift 22T>-)[a]	Mercier et al., 2013 [S4]
SH517	**amyE**::*P*$_{\mathbf{katA}}$**-gfp spc trpC2 pheA1**	Hoover et al., 2010 [S5]
Bss307	**168CA aprE::P$_{\mathbf{rpsD}}$-mcherry spc**	S. Syvertsson, unpublished
4738	**LR2 aprE::P$_{\mathbf{rpsD}}$-mcherry spc**	This study
RM82	**LR2 amyE::P$_{\mathbf{xseB}}$-xseB-ispA spc**	Mercier et al., 2013 [S4]
YK1424	168CA Ω*ispA*::pMutin4-*erm*-*P*$_{spac}$-*ispA*	Mercier et al., 2013 [S4]
YK1494	BS115 **amyE::P$_{\mathbf{xseB}}$-xseB-ispA spc**	This study
YK1450	168CA Ω*hepS*::pMutin4-*erm*-*P*$_{spac}$-*hepS*	This study
YK1522	BS115 *mhqR*::*TnYLB-1* (*kan*)	This study
YK1584	LR2 Ω*zwf*::pMutin4-*erm*-*P*$_{spac}$-*zwf*	This study
YK1604	LR2 Ω*mgsA*::pMutin4-*erm*-*P*$_{spac}$-*bshB1*	This study
YK1694	168CA *xseB*::*TnYLB-1* (*kan*)[b] *amyE*::*P*$_{xyl}$-*accDA spc*	Mercier et al., 2013 [S4]
YK1816	BS115 *ndh*::*TnYLB-1* (*kan*)	This study
YK1817	BS115 *qoxB*::*TnYLB-1* (*kan*)	This study
YK1818	BS115 *ctaB*::*TnYLB-1* (*kan*)	This study
YK1889	168CA Δ*uppS*::*kan* pLOSS-*P*$_{spac}$-*uppS-erm* Ω*P*$_{xyl}$-*cdsA spc*	Kawai et al., 2014 [S6]
YK2003	**BS115 amyE**::*P*$_{\mathbf{katA}}$**-gfp spc**	This study
YK2005	LR2 *aprE*:: **P$_{\mathbf{rpsD}}$**-*mCherry kan amyE*::*P*$_{katA}$-*gfp spc*	This study
YK2027	LR2 Ω*katA*::pMutin4-*erm*-*P*$_{spac}$-*katA*	This study
YK2028	LR2 Ω*sodA*::pMutin4-*erm*-*P*$_{spac}$-*sodA*	This study
E. coli		
TB28	MG1655 **ΔlacIZYA**	Lab. stock
RM345	TB28 **ΔmurA::kan** pOU82-**murA**	Mercier et al., 2014 [S1]
**Plasmid**		
pMutin4	*bla erm P*$_{spac}$ *mcs lacZ lacI*	Vagner et al., 1998 [S7]
pM4-*P*$_{spac}$-*hepS*	*bla erm P*$_{spac}$-*hepS*-5' *lacZ lacI*	This study
pM4-*P*$_{spac}$-*zwf*	*bla erm P*$_{spac}$-*zwf*-5' *lacZ lacI*	This study
pM4-*P*$_{spac}$-*bshB1*	*bla erm P*$_{spac}$-*bshB1*-5' *lacZ lacI*	This study
pM4-*P*$_{spac}$-*katA*	*bla erm P*$_{spac}$-*katA*-5' *lacZ lacI*	This study
pM4-*P*$_{spac}$-*sodA*	*bla erm P*$_{spac}$-*sodA*-5' *lacZ lacI*	This study

cat, chloramphenicol; *tet*, tetracyclin; *erm*, erythromycin; *spc*, specctinomycin; *kan*, kanamycin; *bla*, β-lactamase; represent resistant genes, respectively

[a & b] These mutations repress expression of the *ispA* gene [S4]

Primers

Primer	nucleotide sequence
pM4-*P*$_{spac}$-*hepS*-F	GAA**GAATTC**TGTGAATTTGGGGACAAG
pM4-*P*$_{spac}$-*hepS*-R	GGA**GGATCC**TGGCGGTTTTTGTTTTCG
pM4-*P*$_{spac}$-*zwf*-F	GGG**GAATTC**GGTGTACTAAAATAAAGCTTCG
pM4-*P*$_{spac}$-*zwf*-R	GGG**GGATCC**ACGTCAAACGGGTGATAGTAA
pM4-*P*$_{spac}$-*bshB1*-F	GAA**GAATTC**TTGATCGCGCATGACAAG
pM4-*P*$_{spac}$-*bshB1*-R	GGA**GGATCC**AATGACAAGATCGAGTGC
pM4-*P*$_{spac}$-*katA*-F	GAA**GAATTC**CCAAGAGGTGATAACATGAG
pM4-*P*$_{spac}$-*katA*-R	GGA**GGATCC**CGGCAACTGTTGAGAAACG
pM4-*P*$_{spac}$-*sodA*-F	GAA**GAATTC**CTAAGGAGGAATTATCATGGC
pM4-*P*$_{spac}$-*sodA*-R	GGA**GGATCC**TTTGTGGTTCGCGTGTCCG

Supplemental Experimental Procedures

Bacterial strains, plasmids, primers and growth conditions

The bacterial strains, plasmid constructs and primers for PCR analysis in this study are shown in Supplemental Information. DNA manipulations were carried out using standard methods. *B. subtilis* and *E*. *coli* walled cells were grown on nutrient agar (NA, Oxoid) or in nutrient broth (NB, Oxid) at 30°C. *B*. *subtilis* L-forms and protoplasts were grown in osmoprotective medium composed of 2 x magnesium-sucrose-maleic acid (MSM) pH7 (40 mM $MgCl_2$, 1 M sucrose, and 40 mM maleic acid) mixed 1:1 with 2 x NB or 2 x NA at 30°C. *B*. *subtilis* L-forms and protoplasts were cultured in liquid medium without shaking. *E*. *coli* L-forms were grown on osmoprotective medium composed of 2 x MSM mixed NB with 2% agar at 30°C. Anaerobic growth condition was maintained using anaerobic atmosphere generation bags (AnaeroGen™, Oxid) in an anaerobic jar for growth on plates. Supplements, 1 or 0.5 mM IPTG, 1 or 2% xylose, 5 mM glutathione (Sigma-Aldrich) were added when indicated. When necessary, antibiotics were added to media at the following concentrations: 100 µg/ml ampicillin, 1 µg/ml erythromycin, 5 µg/ml kanamycin, 50 µg/ml spectinomycin, 300 µg/ml, 400 µg/ml D-cycloserine, 400 µg/ml fosfomycin, 200 µg/ml PenG and/or 1 µg/ml 8J (FtsZ inhibitor, [S8]). Experimental Procedures were also described in Supplemental Information.

Construction of IPTG-inducible mutants

The first 200~300 bp of the *hepS*, *zwf*, *bshB1*, *katA* or *sodA* gene containing Shine–Dalgarno sequence was amplified by PCR from genomic DNA of the wild-type strain 168CA using the primers in above table, then cloned between the EcoRI and BamHI sites of plasmid pMutin4 [S7], creating pM4-P_{spac}-*hepS*, *zwf*, *bshB1*, *katA* or *sodA*, respectively (see starin table). The resulting plasmids were introduced into *B*. *subtilis* to generate YK1450, YK1584, YK1604, YK2027 and YK2028, respectively (strain table). In those strains, the full-

length *hepS*, *zwf*, *bshB1*, *katA* or *sodA* gene is expressed from the IPTG-inducible promoter P_{spac}.

Transposon mutagenesis

Transposon mutagenesis was performed essentially as described previously [S9, S10]. To screen for mutants that restored of viability of strain YK1494 (P_{xyl}-*murE amyE*::*ispA*), containing a second copy of *ispA* gene at the *amyE* locus, in the absence of xylose on NA/MSM plates, cells were transformed with the transposon plasmid pMarB and transformants were selected on NA containing 1% xylose at 30°C. Several transformants were picked and individually grown for 8 h in NB with 1% xylose at 30°C. The cells from each culture were then plated and incubated overnight at 50°C on NA plates containing kanamycin and 1% xylose, erythromycin and 1% xylose. We then selected the plate that gave the highest ratio of kanamycin-resistant colonies versus erythromycin-resistant colonies. The selected culture was used to generate a library of about 100,000 colonies on NA containing kanamycin and 1% xylose at 50°C. Mutants that restored the viability to strain YK1494 in the absence of xylose were selected from the library on NA/MSM plates (no xylose) containing 1 µg/ml 8j. Genomic DNA of the mutants were isolated and backcrossed into strain BS115 (P_{xyl}-*murE*) to confirm that the restoration of growth was not due to a second site mutation. Mutants that had stable suppressor mutations linked to a transposon insertion were confirmed by back crossing and were subjected to inverse PCR amplification and sequencing of the transposon insertion site as described previously [S9]. We isolated six independent transposon insertions in *ctaB* or *mhqR*, or two distinct positions of *ndh* or *qoxB*.

Protoplast and L-form preparation in Liquid medium

Exponentially growing *B*. *subtilis* walled cells (OD_{600nm} of 0.2~0.3) in NB/MSM medium with appropriate supplements were harvested and resuspended in fresh NB/MSM containing lysozyme (100 µg/ml) and supplements, if required. The cells were incubated at 37°C with shaking for 1 hr. For protoplast growth (L-form transition), the protoplasts were diluted

(1/1000) into fresh NB/MSM containing supplements, if required, and incubated at 30°C without shaking, as described previously [S4].

RNA isolation

Isolation of total RNA of *B. subtilis* cultures (OD_{600nm} of 0.2~0.3) was carried out as described previously [S11]. Strain BS115 (P_{xyl}-*murE*) was cultured in 10 ml NB/MSM medium with 2% xylose (walled cells). The cells were harvested and resuspended in fresh NB/MSM containing lysozyme (100 μg/ml) with and without 2% xylose. The cell cultures were incubated at 37°C with shaking for 1 hr to generate protoplasts. The protoplast cultures were further incubated in NB/MSM containing 8j and PenG with or without 2% xylose at 30°C for 3 hr or 18 hr. L-forms were prepared by using protoplast of strain LR2 (P_{xyl}-*murE ispA**), as described above.

Microarray analysis

The cDNA synthesis using 5 μg of purified total RNA and the terminal labeling of fragmented cDNAs were performed as described previously [S12]. Hybridization of Genechip *B. subtilis* Array with the labelled cDNA fragments and the scanning of the hybridized Genechip were performed with an appropriate hybridization condition and the program for the array, ProkGE-WS2, according to the manufacture's instruction (Affymetrix). The signal intensities of all probes on Genechip in each hybridization were normalized using the scaling program in GCOS software with a target signal intensity of 500, according to the manufacture's instruction (Affymetrix). On Affymetrix *B. subtilis* array, there are probes corresponding to intergenic regions, gene coding regions (for some genes, there are multiple gene sets) and control probes, then, we used the normarized intensities for the gene coding regions in this analysis. To summarise microarray results in Table 1, we selected the probes corresponding to the genes with adequate signal intensities [sum of the signal intensities of walled cells (P_{xyl}-*murE*, 2% xylose), Protoplasts (P_{xyl}-*murE*, no xylose) of 3 hr and 18 hr incubations, L-

forms (P_{xyl}-*murE ispA**, no xylose) > 400] to eliminate lower expression genes. ArrayExpress accession number is E-MTAB-3380.

Microfluidic system

Microfluidic experiments were carried out using a device described previously [S13]. Briefly, 3 ul of concentrated L-form (or protoplast) culture was added onto the cover glass which formed the bottom of the sample chamber, then a patterned agarose pad was placed (patterned side down) onto the culture in the sample chamber, trapping bacterial cells in the tracks of the agarose pad. The top of the chamber was then sealed with a plasma-treated cover glass (Agar Acientific Ltd, L46s20-5, coverglass 20x20 mm No.5). The assembly was left at room temperature or at 30°C for 20 min to allow plasma bond to set. Patterned agarose pads, with tracks of 1.5 um deep, were cast using an 'Intermediate PDMS mould' with 4% low melting point agarose (SeaPlague GTG Agarose from Lonsza, gelling temp 26–30°C) in L-form medium (NB/MSM/PenG), that set slowly at 30°C for 1 – 2h. The tracks were the repeat of a set of 3 tracks of 0.8 um, 0.9 um and 1.0 um wide, grouped into 15um x 20um blocks divided by gutters. Sample chambers were created by plasma-bonding a PDMS chamber block to a long cover glass (Agar Acientific Ltd, L4239-2, Coverglass 35x64 mm No.1.5). The PDMS chamber block also contained two buffer reservoirs on either side of, and connected to, the sample chamber, one for imputing fresh medium and the other as the outlet of the spent medium and bacterial cells that were not confined in the tracks. Growth medium was supplied continuously through the inlet reservoir from a 50 ml syringe, controlled by a syringe pump at a speed of 3 ml/h using the WinPump Term software (New Era).

Microscopy was performed on a Nikon Eclipse Ti inverted fluorescence microscope system fitted with an Apo TIRF objective (Nikon 60x/1.49 Oil). Light was transmitted from a 300 Watt xenon arc-lamp through a liquid light guide (Sutter Instruments) and images were collected using a HQ2-coolsnap camera (MAG Biosystems). The focus was maintained throughout the experiment using the Perfect Focus System (Nikon). All filters were Modified

Magnetron ET Sets from Chroma. The filter for GFP was 49002 ET-EGFP (FITC/CY2) (exciter ET470/40x; dichroicT495LP; emitter ET525/50M). For mCherry the filter used was 49005 ET-DsRed (TRITC/Cy3) (exciter ET545/30x; dichroicT570LP; emitter ET620/60m). Digital images were acquired and analysed using Frap-AI 7.7.5.0 (MAG Biosystems).

Lipid peroxidation

Lipid oxidation was detected using a fluorescent probe (C_{11}-BODIPY$^{581/591}$; Molecular Probes) as described previously [S14-S16] . Exponentially growing *B*. *subtilis* walled cells were cultured in NB/MSM with or without 1 mM H_2O_2 at 37°C for 1 hr, and then 5 μM C_{11}-BODIPY$^{581/591}$ was added to the culture and incubated for 1 hr at 30°C. The cells were used for microscopic analysis. For protoplasts and L-forms, 5 μM C_{11}-BODIPY$^{581/591}$ was added to overnight cultures and the cultures were incubated for 1 hr at 30°C before being used for microscopic analysis.

References

S1. Mercier, R., Kawai, Y., and Errington, J. (2014). General principles for the formation and proliferation of a wall-free (L-form) state in bacteria. eLife *3*.

S2. Nakano, M.M., and Zuber, P. (1998). Anaerobic growth of a “strict aerobe” (*Bacillus subtilis*). Annu. Rev. Microbiol. *52*, 165-190.

S3. Leaver, M., Dominguez-Cuevas, P., Coxhead, J.M., Daniel, R.A., and Errington, J. (2009). Life without a wall or division machine in *Bacillus subtilis*. Nature *457*, 849-853.

S4. Mercier, R., Kawai, Y., and Errington, J. (2013). Excess membrane synthesis drives a primitive mode of cell proliferation. Cell *152*, 997-1007.

S5. Hoover, S.E., Xu, W., Xiao, W., and Burkholder, W.F. (2010). Changes in DnaA-dependent gene expression contribute to the transcriptional and developmental

response of *Bacillus subtilis* to manganese limitation in Luria-Bertani medium. J. Bacteriol. *192*, 3915-3924.

S6. Kawai, Y., Mercier, R., and Errington, J. (2014). Bacterial cell morphogenesis does not require a preexisting template structure. Curr. Biol. *24*, 863-867.

S7. Vagner, V., Dervyn, E., and Ehrlich, S.D. (1998). A vector for systematic gene inactivation in *Bacillus subtilis*. Microbiol. *144*, 3097-3104.

S8. Adams, D.W., Wu, L.J., Czaplewski, L.G., and Errington, J. (2011). Multiple effects of benzamide antibiotics on FtsZ function. Mol. Microbiol. *80*, 68-84.

S9. Le Breton, Y., Mohapatra, N.P., and Haldenwang, W.G. (2006). In vivo random mutagenesis of *Bacillus subtilis* by use of TnYLB-1, a mariner-based transposon. Appl. Environ. Microbiol. *72*, 327-333.

S10. Kawai, Y., Daniel, R.A., and Errington, J. (2009). Regulation of cell wall morphogenesis in *Bacillus subtilis* by recruitment of PBP1 to the MreB helix. Mol. Microbiol. *71*, 1131-1144.

S11. Dominguez-Cuevas, P., Mercier, R., Leaver, M., Kawai, Y., and Errington, J. (2012). The rod to L-form transition of *Bacillus subtilis* is limited by a requirement for the protoplast to escape from the cell wall sacculus. Mol Microbiol *83*, 52-66.

S12. Kusuya, Y., Kurokawa, K., Ishikawa, S., Ogasawara, N., and Oshima, T. (2011). Transcription factor GreA contributes to resolving promoter-proximal pausing of RNA polymerase in *Bacillus subtilis* cells. J. Bacteriol. *193*, 3090-3099.

S13. Moffitt, J.R., Lee, J.B., and Cluzel, P. (2012). The single-cell chemostat: an agarose-based, microfluidic device for high-throughput, single-cell studies of bacteria and bacterial communities. Lab on a chip *12*, 1487-1494.

S14. Drummen, G.P., van Liebergen, L.C., Op den Kamp, J.A., and Post, J.A. (2002). C11-BODIPY(581/591), an oxidation-sensitive fluorescent lipid peroxidation probe: (micro) spectroscopic characterization and validation of methodology. Free Radical Bio. Med. *33*, 473-490.

S15. Johnson, L., Mulcahy, H., Kanevets, U., Shi, Y., and Lewenza, S. (2012). Surface-localized spermidine protects the *Pseudomonas aeruginosa* outer membrane from antibiotic treatment and oxidative stress. J. Bacteriol. *194*, 813-826.

S16. Pap, E.H., Drummen, G.P., Winter, V.J., Kooij, T.W., Rijken, P., Wirtz, K.W., Op den Kamp, J.A., Hage, W.J., and Post, J.A. (1999). Ratio-fluorescence microscopy of lipid oxidation in living cells using C11-BODIPY(581/591). FEBS Lett. *453*, 278-282.
